# The diagnostic indicators of gestational diabetes mellitus from second trimester to birth: a systematic review

**DOI:** 10.1186/s40842-021-00126-7

**Published:** 2021-10-11

**Authors:** Daria Di Filippo, Thiyasha Wanniarachchi, Daniel Wei, Jennifer J. Yang, Aoife Mc Sweeney, Alys Havard, Amanda Henry, Alec Welsh

**Affiliations:** 1 School, of Women’s and Children’s Health, University of New South Wales, Sydney, NSW Australia; 2 Faculty of Medicine, University of New South Wales, Sydney, NSW Australia; 3 Department of Women’s and Children’s Health, St George Hospital, Sydney, NSW Australia; 4 National Drug and Alcohol Research Centre - Faculty of Medicine, University of New South Wales, Sydney, NSW Australia; 5 Centre for Big Data Research in Health - Faculty of Medicine, University of New South Wales, Sydney, NSW Australia; 6 Department of Maternal-Fetal Medicine, Royal Hospital for Women, Locked Bag 2000, Barker Street, Randwick, NSW 2031 Australia

**Keywords:** Gestational diabetes mellitus, Diagnosis, Biomarker, Indicators

## Abstract

**Background:**

Gestational diabetes mellitus (GDM) is glucose intolerance first recognised during pregnancy. Both modalities and thresholds of the GDM diagnostic test, the Oral Glucose Tolerance Test (OGTT), have varied widely over time and among countries. Additionally, OGTT limitations include inconsistency, poor patient tolerability, and questionable diagnostic reliability. Many biological parameters have been reported to be modified by GDM and could potentially be used as diagnostic indicators. This study aimed to 1) systematically explore biomarkers reported in the literature as differentiating GDM from healthy pregnancies 2) screen those indicators assessed against OGTT to propose OGTT alternatives.

**Main body:**

A systematic review of GDM diagnostic indicators was performed according to PRISMA guidelines (PROSPERO registration CRD42020145499). Inclusion criteria were full-text, comprehensible English-language articles published January 2009-January 2021, where a biomarker (from blood, ultrasound, amniotic fluid, placenta) was compared between GDM and normal glucose tolerance (NGT) women from the second trimester onward to immediately postpartum. GDM diagnostic method had to be clearly specified, and the number of patients per study higher than 30 in total or 15 per group. Results were synthesised by biomarkers.

**Results:**

Of 13,133 studies identified in initial screening, 174 studies (135,801 participants) were included. One hundred and twenty-nine studies described blood analytes, one amniotic fluid analytes, 27 ultrasound features, 17 post-natal features. Among the biomarkers evaluated in exploratory studies, Adiponectin, AFABP, Betatrophin, CRP, Cystatin-C, Delta-Neutrophil Index, GGT, TNF-A were those demonstrating statistically and clinically significant differences in substantial cohorts of patients (> 500). Regarding biomarkers assessed versus OGTT (i.e. potential OGTT alternatives) most promising were Leptin > 48.5 ng/ml, Ficolin3/adiponectin ratio ≥ 1.06, Chemerin/FABP > 0.71, and Ultrasound Gestational Diabetes Score > 4. These all demonstrated sensitivity and specificity > 80% in adequate sample sizes (> / = 100).

**Conclusions:**

Numerous biomarkers may differentiate GDM from normoglycaemic pregnancy. Given the limitations of the OGTT and the lack of a gold standard for GDM diagnosis, advanced phase studies are needed to triangulate the most promising biomarkers. Further studies are also recommended to assess the sensitivity and specificity of promising biomarkers not yet assessed against OGTT.

**Trial registration:**

PROSPERO registration number CRD42020145499.

**Supplementary Information:**

The online version contains supplementary material available at 10.1186/s40842-021-00126-7.

## Background

In Gestational Diabetes Mellitus (GDM) the pregnancy-related physiological impairment of glycaemic control and insulin resistance are such that the mother, and consequently the fetus, are exposed to glycaemic levels considered diagnostic of diabetes [1]. GDM is defined internationally as “Hyperglycaemia first recognized during pregnancy” [2], refined in 2015 by the American Diabetes Association (ADA) as “diabetes diagnosed in the second and third trimesters of pregnancy” [3]. Methods and thresholds to identify GDM in pregnancy have changed several times in the last 50 years; currently the most common is the oral glucose tolerance test (OGTT), where 75 g of glucose are ingested by women after an overnight fast and Blood Glucose Level (BGL) is checked at zero, one and two hours after ingestion [4]. Most commonly this is performed at 24–28 weeks gestation, or in case of high-risk patients at 12–16 weeks and again at 24–28 weeks if the initial test is normal.

Initially, GDM was only diagnosed at glycaemic levels that would be considered diagnostic of Type 2 diabetes mellitus in non-pregnant adults. Subsequent studies, including the Australian Carbohydrate Intolerance Study in Pregnant Women (ACHOIS) and the Hyperglycemia and Adverse Pregnancy Outcome (HAPO) study, have demonstrated that lower levels of glycaemia may still be associated with adverse maternal or fetal outcome [5, 6], so GDM diagnostic thresholds have been progressively lowered. The current most used criteria worldwide are those released by IADPSG (The International Association of Diabetes and Pregnancy Study Groups): Fasting: 92 mg/dL (5.1 mmol/L), one hour: 180 mg/dL (10.0 mmol/L), two hours: 153 mg/dL (8.5 mmol/L) [2].

The reliability of OGTT has been questioned, as it involves a supra-physiological load unrelated to body weight or normal dietary intake. As well as being unpleasant, expensive and time consuming, OGTT has poor reproducibility: up to 30% of patients with positive screening results screen negative when re-tested [7–11].

While the deficiencies of the OGTT are clear, any replacement is hampered by the lack of a true gold standard for GDM diagnosis. Simply comparing new methods against the poorly reliable OGTT will fail to uncover false-positive and false-negative screening misclassifications. We undertook a systematic review of all the biochemical, clinical and pathological parameters proposed to be altered by GDM in order to aid with identification of a biomarker that accurately differentiates between women who do and do not develop GDM.

The objectives of this study were therefore:

1. To systematically review the literature on biomarkers assessed for their ability to differentiate GDM from NGT pregnancies.
2. To describe characteristics, methodological quality, and findings of studies assessing biomarkers for their predictive accuracy versus OGTT, thereby identifying the most promising to use to effectively discriminate between GDM and NGT pregnancy as compared to the current diagnostic method of OGTT.

## Main text

### Methods

We conducted a systematic review of biomarkers of Gestational Diabetes in accordance with PRISMA guidelines (Additional file 2).

#### Eligibility criteria (PICOS details summarised in Table 1)

**Table 1:** Inclusion and exclusion criteria

Criteria	Inclusion	Exclusion
Population	Pregnant women classified as having or not GDM with a specified method	Women with diabetes and other pregnancy complications such as preeclampsia or hypertension, not valid comparison, not specified diabetes type, studies in animals
Intervention	Evaluation of blood analytes, amniotic fluid analytes, annexes samples, and ultrasound assessment Analytes collected from the 14^th^ weeks of gestation to the time of birth and focused on GDM women	Genetic analytes (e.g. nucleotide polymorphisms), urine analytes
Comparison	Non GDM patients or different groups of GDM patients (for examples diet only, medications)	
Outcomes	- Comparison of values between GDM patients compared to non GDM ones (measured/compared and reported) - A total cohort of at least 30 AND/OR groups of minimum 15 patients and specified diagnostic process	
Study design	Randomized control trial, case–control, cohort, diagnostic study Comprehensible English language	Systematic reviews, case-reports, review articles
Quality assessment	CASP scores: at least 7/11 for case–control studies and 8/12 for diagnostic and cohort studies	

*CASP* Critical Appraisal Skills Programme

*Annexes sample* Biomarkers of placenta and umbilical cord

Full-text articles (conference abstracts only excluded), written in comprehensible English and published January 2009—January 2021. Only randomized controlled trial (RCT), case–control or cohort studies (retrospective or prospective) were accepted, excluding systematic reviews, case report studies, letters. They had to describe biomarkers (blood, ultrasound, placenta/umbilical cord) measured from the second trimester (14 weeks gestation) of human pregnancy to the delivery period (within one-hour post-partum), and values described in GDM women versus NGT, and/or within subgroups of the GDM patients (e.g. diet treated only vs medication). Authors must have specified the method and thresholds used to diagnose GDM and the number of patients included in the study had to be higher than 30 in total or 15 per subgroup. Articles reporting genetic tests (e.g. methylation or miRNA expression) were excluded, as our aim was to identify an inexpensive biomarker that could accurately diagnose GDM worldwide.

##### Additional inclusion criteria

For the second aim of our study, the indicators must have been compared to OGTT, reporting at least sensitivity and specificity.

#### Data sources, search strategy and additional articles identification

The initial search was run on the 15/05/2019, and a final/updated search was performed on the 07/02/2021. Six databases were screened: EBM, Medline, Embase, Cochrane, Web of Science, Scopus using keywords “Gestational diabet*” or “pregnancy diabetes” or “GDM” AND “marker” or “biomarker” or “diagnos*” or “indicator”. The time limit was set for publications to be from 01.01.2009 to focus on recent evidence. The references of 20% of the included articles were reviewed to confirm that our search identified the majority of relevant articles.

#### Screening and data extraction

Initial screening of the titles and abstracts of articles returned by the database search was performed by DDF and TW. The screening for the update was performed by DDF, TW, DW, AM. Papers for potential inclusion were then read in full by DDF and a second co-author (TW, DW, JY, AM) to check eligibility. Disputes regarding article inclusion were resolved by joint senior author AH.

Once considered eligible, articles were downloaded and read before re-checking eligibility with an inclusion criteria checklist: if included, a Data Extraction Form (Additional file 3) was completed together with a CASP (Critical Appraisals Skill Programme) form and CASP table for quality assessment. Each article was double reviewed. Quality assessment of included articles was conducted by DDF and a second reviewer using the CASP checklists available for each type of article: Diagnostic, RCT, cohort and case–control [12]. Articles insufficiently fulfilling CASP criteria (< 7/11 for case–control and RCT and < 8/12 for diagnostic studies and cohort studies) were excluded.

##### Data collection process and items

Data were sought about authors’ names, country of study, aim of the study, inclusion/exclusion criteria for participants, method used to diagnose GDM, gestational age at collection, number of cases/controls, settings (ambulatory/delivery room), markers studied, research design (case–control, cohort, diagnostic, randomised control trial), methods, summary of findings, conclusions.

##### Risk of bias in individual and across studies

We assessed the risk of bias in individual studies by using the CASP checklists appropriate to the type of studies included: case–control, cohort, RCT, diagnostic; the latter reporting the sensitivity and specificity of the indicator as assessed against OGTT.

##### Summary measure and additional analysis

If available, a risk ratio or difference in mean was reported. When described, the sensitivity and specificity of the biomarker assessed against OGTT, and if possible, the number of OGTT that could be avoided by using the indicators were noted. For articles reporting the assessment of a biomarker against OGTT, we used the CASP for diagnostic study checklist. Given the heterogeneous nature of the studies included in terms of GDM diagnostic criteria, definitions of cases and controls, biomarker used, and time in pregnancy of sample collection, a formal meta-analysis was not appropriate, so results are presented as narrative synthesis in tabular form. Articles were classified based on the Weinstein et al. classification [13]. Studies assessing only a difference of values between GDM and NGT patients were considered as “Exploratory phase” (defined as substantial if cohort > 500 patients). Studies reporting the biomarker’s sensitivity and/or specificity compared to OGTT were considered as “Challenge phase” (small when < 100 patients’ sample and adequate when > 100 patients). The results of the challenge phase were considered good with sensitivity and specificity > 80% and very good with sensitivity and specificity > 90%. Based on the phase identified for each article, we then aimed to propose the next phase for further assessing each biomarker: “Challenge phase” to test them against OGTT or “Advanced phase” to confirm and further explore the results of the challenge phase studies.

### Results

#### Characteristics of included studies

**Fig. 1:**
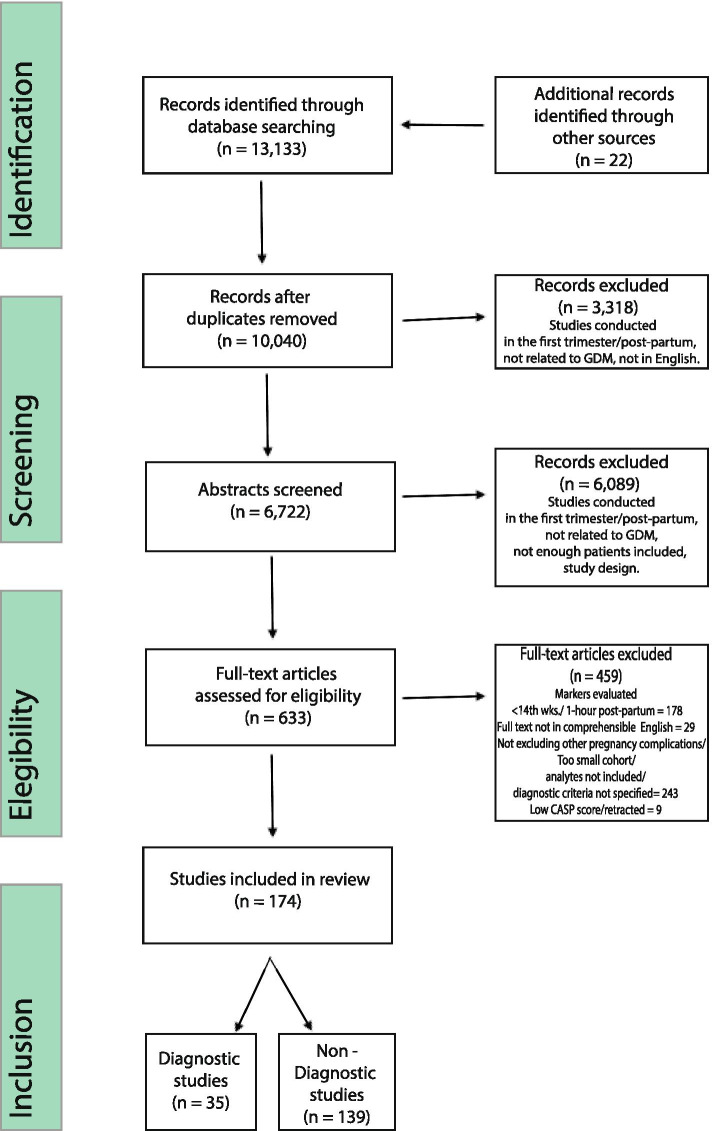
Screening and selection process

Of the 13,133 titles and abstract examined (22 of which were identified through reference list searches), 634 full-text articles were assessed, and 174 articles including 135,801 participants were included (Fig. 1).

The most common reason for exclusion was assessment of biomarkers in the first trimester or later than 1-h post-partum. The number of participants in each study ranged from 35 [14] to 4926 [15].

**Fig. 2:**
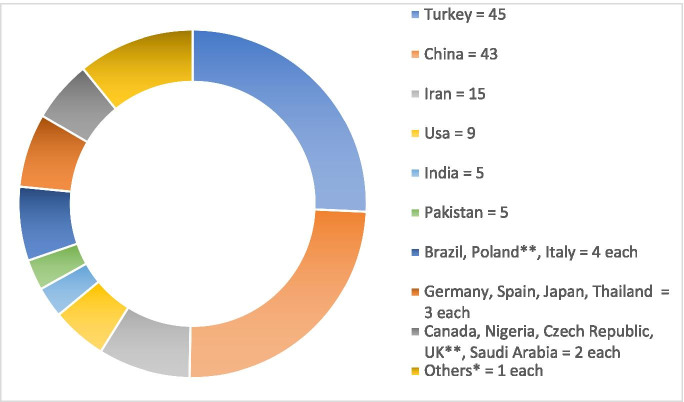
Location of included studies. *Others = Switzerland, Austria, Hungary, Greece, Romany, Serbia, Israel, United Arab Emirates, Sri Lanka, South Africa, Sudan, Mexico, Canada, Bangladesh, Hong Kong, Malaysia, Korea, New Zealand, Australia; ** One study held in both UK and Poland

Regarding methodology, 40 included articles described cohort studies (31 prospective and nine retrospective) and 134 case–control studies. Thirty-five included articles were considered diagnostic as they assessed diagnostic potential of the biomarkers against OGTT, specifying a cut-off. Regarding study location, most studies were conducted in Turkey (45) and China (43) (Fig. 2).

Diagnostic criteria for GDM were heterogeneous; most studies used the 2010 IADPSG criteria [2] adopted by the ADA in 2011 and the World Health Organization (WHO) in 2013 (Table 2). These criteria were used in 100 studies (of which 3 used 50 g Glucose Challenge Test (GCT) as an initial screening test). The second most common criteria were the Carpenter and Coustan’s criteria (C&C) [16], released in 1982 and adopted by ADA until 2011, used in articles written up to 2020. The remaining articles followed the WHO criteria [17], the National Diabetes Data Group (NDDG) criteria [18] or local guidelines.

**Table 2:** Diagnostic criteria for gestational diabetes mellitus: details and usage

Name and year	Articles adopting the criteria (publications’ time frame)^a^	Steps	Glucose load (grams)	FGL	BGL 1	BGL 2	BGL 3	Abnormal values needed
*NDDG* 1979	12 (2014 to 2020)	2	50—100	5.8	10.6	9.2	8.0	2
*C&C* 1982 (ADA < 2011)^b^	49 (2009 to 2020)	2	50—100	5.3	10	8.6	7.8	2
*WHO* 1999	13 (2009 to 2018)^c^	1	75	7.0	X	7.8	X	1
*IADPSG* 2010 (ADA > 2011)^b^ (WHO > 2013)^b^	100 (2012 to 2020)	1	75	5.1	10	8.5	X	1
Others	6							
NICE	2 (2019–2020)	1	75	5.6	X	7.8	X	1
*EASD* 1991	1 (2014)	1	75	6.0	X	9	X	1
*ADIPS* 2014	1 (2014 to 2015)	1	75	5.5	X	8	X	1
*JSOG* 2002	1 (2010–2020)	2	50 – 75	5.5	10	8.3	X	1

*NDDG* National Diabetes Data Group, *C&C* Carpenter and Coustan, *ADA* American Diabetes Association, *WHO* World Health Organization, *IADPSG* International Association of Diabetes in Pregnancy Study Groups, *EASD* European Association for the Study of Diabetes, *ADIPS* Australasian Diabetes in Pregnancy Society, *JSOG* Japan Society of Obstetrics and Gynaecology, *X* Not applicable, *FGL* Fasting Glucose Level, *BGL* Blood Glucose Level

^a^ 7 articles used more than 1 criteria over years

^b^ association in brackets accepted the criteria before/after the specified year

^c^ 2 articles used modified WHO 99 criteria = 75 gr OGTT fasting: 0h < 5.3/5.6 mm/l; 1 h < 10.6/8.9 mm/l; 2 h < 8.9/7.8 mm/l, one used the WHO 97 criteria as adapted locally in Birdem – Bangladesh

Among the 174 included articles, 129 described maternal blood analytes, one reported amniotic fluid analytes, 27 described ultrasound features of the mothers, the fetuses, the placenta/umbilical cord or a combination of ultrasound features, and 17 assessed postnatal features of the babies and the placentas.

Most biomarkers were assessed only once during pregnancy between the 14^th^ and the 41^st^ week of gestational age. Twelve articles reported multiple biomarker assessment timepoints: ten studies evaluated biomarkers two times [19–28], and two on three occasions [29, 30]. More than 150 biomarkers were evaluated, as listed in full in Additional file 4. The most assessed category among blood and amniotic fluid biomarkers was proteins (41), followed by score/ratio/indices/miscellaneous derived markers (20), cytokines (13), hormones (13) and oxidation/peroxidation products (11). The remaining categories were represented by advanced glycation end products, enzyme inhibitors, peptides and amino acids, lipid molecules and lipid-derived products, growth factor, vitamins, and other organic compounds.

#### Synthesis of results

The results of individual studies separated into the different types of biomarkers are detailed in Additional file 1, reported as GDM vs NGT and with significance at a *p* value < 0.05, unless otherwise stated.

##### Haematological (Additional file 1a)

There were six different categories of haematological biomarkers. The first category refers to variations of OGTT/ glycation end products/ full blood count features. Fasting blood glucose (FBG) was described to be more frequently higher than 91.8 mg/dL in GDM (*n* = 33,466) [15, 31, 32]. Among the Glycation End products, Gosh et al. reported median pGCD59 to be tenfold higher in GDM (*n* = 627, 3.23 vs 0.33 SPU) [33], and HbA1c was reported to be higher in seven articles (*n* = 21,181), (29–43.2 vs 26.8–35.5 mmol/L) [34–40] Regarding full blood count features, Celtik et al. [41] reported significantly higher Mean Platelet Volume (MPV) in GDM (*n*= 145; 8.66 vs 8.27 FL).

The second category of Additional file 1a describes lipids, metabolism markers, adipose tissue markers and adipokines. Four articles (*n* = 251) demonstrated use of metabolomics analysis to differentiate GDM patients [42–45]. Several studies found higher lipids in GDM: total cholesterol [23, 46],triglycerides [47, 48]; low and very low-density lipids [46, 49, 50] and triacylglycerol [46]. Nar et al. [51] (*n* = 129), in contrast, found no significant differences in lipid parameters. Among the metabolism markers, Yuan et al. in two articles [29, 52] (*n* = 359) reported significantly increased Ficolin-3, Ficolin-3/Adiponectin ratio, and secreted frizzled-related protein 4 (SFRP4) (*n* = 359, 10.64 lg/mL vs 8.24 lg/mL, 1.26 vs 0.93 and 12.84 *vs* 10.17 ng/mL respectively). Among the adipokines, Adiponectin was reported as lower in GDM by several studies (total n > 1,200) as were Adropin levels (*n* = 60, 1.5 vs 3.3 ng/mL) [53], Vaspin (*n* = 237, 1.31 vs 1.69 ng/mL) [54] and Follistatin (*n* = 277, 8.216.32 vs 9.2263.41, ng/mL) [55]. Adipocite Fatty Acid Binding Protein (AFABP) was reported to be significantly higher in four studies (*n* = 684) [21, 56–58], as was Retinol-Binding Protein 4 in two (*n* = 305) [56, 59]. Leptin was significantly higher in five studies [20, 21, 58, 60, 61] (*n* = 1148), however not different for Sengul et al. [62] (*n* = 40).

The third category of Additional file 1a describes Hormones and their transport molecules, Growth Factors, peptides, Vitamins, and Iron Studies. Betatrophin was reported to be higher in GDM in three studies (*n* = 634) [63–65], as was Unconjugated Estriol (*n* = 523, 1.15 vs 1.05 multiples of the median) [66]. Sex Hormone Binding Globulin (SHBG) was lower in two studies (*n* = 140) [67, 68] as was Copeptin (3.5 vs 4.4 pmol/l, *p* < 0.05) [69]. In terms of Growth Factors, VEGF levels were described as increased [61]. Among the peptides, Galanin was found to be higher in GDM in three studies [67, 70, 71], while ANP and BNP were decreased (12.9 vs 34.8 pg/ml, and 416.6 vs 629.7 mg/dl, respectively). Of the vitamins, 25 OH-D was significantly reduced in GDM (*n* = 310, 13.0- 13.9 vs 17.5–17.6 ng/mL) [72, 73]. Afkhami-Ardekani [74] described higher concentrations of transferrin saturation (26.49 vs 12.77) and ferritin (73.34 vs. 41.55 ng/ml), and lower Total Iron Binding capacity (383.09 vs 457.79 μg/dl) [74].

The fourth category of haematological biomarkers describes Oxidative Stress, Antioxidants, Inflammation, and Immune System markers. 8-Isoprostane was significantly higher in GDM as reported by two studies (*n* = 272): Rueangdetnarong et al. in the second trimester (737.5 vs 249.1 ng/mL) and early labour (666.4 vs 104.8 ng/mL) [22] and Shang et al. in the third trimester [60]. Regarding inflammatory markers, CRP was found to be similar in GDM versus controls in three papers (*n* = 195) [74–76] and higher in seven (*n* = 1,364) [26, 77–82]. IL-2, IL-6 and IL-18 were all reported as increased in GDM, as was TNF–A in the majority of papers, (*n* = 821) [22, 75, 83–86] and reported as similar in concentration to controls in three studies (*n* = 167) [78, 87]. Among the immune system biomarkers, Delta Neutrophil Index and Neopterin levels were described as higher in GDM (*n* = 728, -2.3 vs -3.0 and *n* = 119, 5.3 vs 3.8 nmol/l) [88, 89].

The fifth category reports pancreas, liver, and kidney biomarkers. Preptin was higher in GDM (*n* = 45, 446.33 vs 157.26 pg/mL) [23], as was Secreted Frizzled-Related Protein 4 (*n* = 359, 12.84 *vs* 10.17 ng/mL) [29] and Pancreatic-derived factor (PANDER) (*n* = 80, 448.0 vs 140.1) [90]. Fasting Insulin, Insulin-to-glucose ratio, Homeostasis Model Assessment Insulin (HOMA), Homeostasis Model Assessment-Insulin Resistance (HOMA-IR), HOMA-B and QUICKI were all reported as significantly increased by several studies (*n* = 752) [49, 91, 92]. Regarding liver function markers, Gamma GT was reported as higher in GDM in two studies (*n* = 2,670) [70, 93]. Among the renal function markers, Cystatin C levels were reported to be higher in two studies (*n* = 552) [94, 95] and in similar concentrations by Yousefzadeh et al. [47] (*n* = 60).

The final category of haematological biomarkers includes musculoskeletal, cardiovascular, endothelial, adhesion molecules and placental biomarkers. Among the musculoskeletal biomarkers, C–Telopeptide X Crosslaps were found to be higher, whereas Osteopontin (*n* = 78) [96], Irisin (*n* = 298) [97–99] and Rank-L (*n* = 92) [87] lower. Among the Cardiovascular factors, Angiopoietin-related growth factor (AGF) levels were higher (*n* = 77) [100], as were Ischemia Modified Albumin [24], Urotensin II, Trimethylamine-*N*-Oxide, Pigment Epithelium Derived Factor [101], and Coagulation Factors [102]. Of the Adhesion Molecules, Vascular adhesion protein 1 (VAP-1) was found to be higher in GDM (*n* = 135, 3.3 vs 1.2 ng/mL) [103]. Lastly, Placental Growth Factor was increased in GDM (*n* = 158, 0.2 vs 0.1 pg/mL, *p* = 0.029) [104].

Two articles calculated a combination of factors/haematological ratios: the first [81] (*n* = 792) described HbA1c and hs-CRP being higher and SHBG lower in women who developed GDM; in the second [82] (*n* = 100) HBA1c and CRP were higher and SHBG and PAPP-A lower in GDM.

##### Amniotic fluid biomarkers (Additional file 1b)

There were two groups of amniotic fluid biomarkers described by Melekoglu et al. [105] (*n* = 40) with increased levels of ADAMTS4 and ADAMTS5 in GDM (Table 3b). These are markers of alterations in the extracellular matrix and abnormal placentation in response to the increase of inflammatory mediators such as IL-6 and TNF-a.

##### Ultrasound biomarkers (Additional file 1c)

There were 4 types of ultrasound biomarkers: maternal, fetal, annexes and combined. In the *maternal section*, the epicardial fat thickness of both mothers and babies was higher in GDM in the study of D’ambrosi (*n* = 168) [106] and Yavuz et al. [107] and by Nar et al. [51] (*n* = 209). An increase in mean subcutaneous adipose thickness was found in GDM by both D’ambrosi [106] and Kansu-Celik [108]. Among the cardiovascular features, Tosun et al. [109] report significant differences in the superior mesenteric artery doppler systolic/diastolic ratio and the resistance index in GDM women, both increased. Isovolumic relaxation time (IRT) was reported by Nar et al. and Aguilera et al. as significantly higher (75–80.8 vs 68–71.6 ms [51, 110] (*n* = 773).

In the *fetal* section, asymmetrical macrosomia, as well as increased fetal liver volume were reported to be more frequent by Ilhan (*n* = 97) [111]. Fetal abdominal wall thickness was increased in three studies (*n* = 490) [14, 112, 113], with one also finding increased maximum subcutaneous fat tissue thickness at the head circumference and thoracic spine levels [14]. A retrospective cohort study on 44,179 women, found no differences in terms of Head Circumference, Femur Length and Estimated Fetal Weight in pregnancy with and without GDM [114]. Epicardial fat thickness was described as significantly greater by Yavuz et al. and Aydin et al. (*n* = 200), albeit a very small absolute difference (1.34 vs 1.31 mm and 1.0 vs 0.8 mm respectively) [107, 115].

In the *annexes* section, To et al. [116] reported the diameter and the mean flow volume of the UV to differ in GDM (*n* = 78, 8.23 vs 2.29 mm, *p* = 0.001 and 8.16 vs 7.54 cm/s, *p* = 0.03, respectively). Lastly, among the combined biomarkers, Perovic [117] (*n* = 110) proposed an Ultrasound Gestational Diabetes Score (UGDS) based on the combination of maternal, fetal and annexes features, that was increased in GDM.

##### Neonatal, umbilical cord and placental biomarkers (Additional file 1d)

Cord blood Estradiol [118] (*n* = 408, 44.1 vs 49.9 nmol/L, *p* = 0.032), as well as Adropin [53] (*n* = 60, 1.5 vs 3.3 ng/mL, p < 0.001), were reported to be significantly lower. The levels of C-Peptide, Glucose levels and Neopterin were found to be higher in newborns of women with GDM by Ipekci [88]. Among the placental inflammatory markers, CD163 and Iron were reported to be higher as was Cyclophilin-A [119] (*n* = 43). Placental weight was significantly higher in GDM in three studies [61, 120, 121], with Kukuc et al. [121] also reporting increased Placenta Weight/Birth Weight ratio, whereas Pooransari reported no significant differences [122]. Dairi et al. [123] and Kadivar et al. (*n* = 306) [124] demonstrated altered placental villous histological morphology in GDM and meconium-laden macrophages were found in greater concentration by Barke et al. [125].

Regarding biomarkers assessed multiple times during pregnancy, most of them were found to increase during pregnancy either exclusively or more often in GDM vs NGT, especially those related to inflammation (TNF-A, IL-10, CRP) [22, 26], per/oxidation (PCO, AOPP, 8ISO) [30], lipid concentration (TC, LDL, V-LDL, TG) [26], vascular damage (TMAO) [25] and metabolism (Preptin, Leptin, AFABP, LCPUFAs) [19, 21, 23]. Some metabolism (Adiponectin) [21] and vascular damage biomarkers (IMA) decreased during GDM as opposed to NGT pregnancies [24].

#### Additional analysis

##### Challenge phase studies

A total of 35 studies (*n* = 61,949) assessed biomarkers for the ability to predict OGTT results (Table 3): 30 haematological, of which two used multiparametric prediction models, and five ultrasound features, of which one used ultrasound multiparametric modelling.

Anjalaski et al. assessed the use of non-fasting OGTT in 800 women, reporting no statistically significant difference with fasting OGTT [126].Hba1c and FBG were most frequently evaluated, but these did not yield good sensitivity and specificity at any threshold examined. HbA1C demonstrated wide ranges of sensitivity (7–91.3%) and specificity (3%-100%) in seven articles [34–40]. FBG, assessed in three articles, had a higher maximum sensitivity and a lower maximum specificity than Hba1c (sensitivity 78.5–96% and specificity 55–78.4%) [15, 31, 32]. Among the biomarkers with very good sensitivity and specificity (> 90%), Leptin > 48.5 ng/ml [20] and Ficolin-3/adiponectin ratio ≥ 1.06 [52] were assessed on sizeable samples (*n* = 508 and 360 respectively) and can be evaluated in advanced phase studies. Lysophospholipids [42], and SHBG (> 50 nmol/L) [68] (*n* = 50 and 90 respectively), however, need to be confirmed in bigger challenge phase studies. Both the composite indices had moderate sensitivity: 69% [81] and 75% [82] respectively.

On ultrasound, subcutaneous fat tissue thickness was evaluated against OGTT. Celik et al. (*n* = 223) [108] found low sensitivity and specificity of this measurement in mothers whereas Tantanasis et al. [14] found 100% sensitivity and specificity in a small cohort of 35 fetuses (for values of 3.9 mm at Head Circumference, 4.5 mm at Abdominal Circumference and 4.7 mm at Thoracic Spine Level). Lastly, Perovic reported a sensitivity of 91% and specificity of 90% for the UGDS score in 100 patients, that hence needs to be assessed in Advanced phase studies [117].

**Table 3:** Challenge phase studies (CPS) details: sensibility/sensitivity results and need of Advanced Phase studies (APN)

Author & Year	Ref N	Design: N of patients	OGTT Criteria	Marker and thresholds	Sensitivity	Specificity	CASP Score	Notes^a^
Trujillo 2014	[15]	CC: 4040 NGT, 886 GDM	IADPSG	Fasting blood glucose > A = 80, B = 85 mg/dl	A = 97% B = 93%,	A = 55% B = 78%	9	A/C
Ruetschi 2016	[31]	COH: 2047 NGT, 251 GDM	IADPSG	Fasting blood glucose > 4.4 mmol⁄ l	79%	69%	8	C/C
D’emden 2020	[32]	COH: 3946 GDM, 22,296 NGT	IADPSG	Fasting blood glucose > 4.6 mmol⁄ l	54%	77%	8	C/C
Anjalakshi 2009	[126]	COH: 713 NGT, 87 GDM	WHO 99	Non fasting OGTT	100%	100%	8	A/A: APN
Kwon 2015	[35]	COH: 242 GDM,79 NGT	C&C	Glycated haemoglobin (HbA1C) > A = 5.05, B = 5.25%	A = 91% B = 74%	A = 62% B = 77%	9	A/C C/C
Renz 2015	[36]	COH: 176 NGT,86 GDM	C&C /IADPSG	Glycated haemoglobin (HbA1C) > A = 6.5, B = 5.8, C = 5.0%	A = 7% B = 26% C = 90%	A = 100% B = 95% C = 33%	11	D/A D/A A/D
Rajput 2012	[34]	COH: 43 GDM, 560 NGT	C&C /IADPSG	Glycated haemoglobin (HbA1C) > A = 5.95, B = 5.45,C = 5.45—5.95%,	A = 29% B = 86% C = 86%	A = 97% B = 61% C = 3%	9	B/C
Siricharoenthai 2020	[37]	CC: 35 GDM, 79 NGT	NDDG	Glycated haemoglobin (HbA1C) > 5.8%	17%	100%	8	D/A
Khan 2020	[38]	P. COH: 50 GDM, 230 NGT	IADPSG	Glycated haemoglobin (HbA1C) > 6.06%	70%	84.8%	8	C/B
Lai 2020	[40]	R.COH: 3547 GDM, 15,714 NGT	IADPSG	Glycated haemoglobin (HbA1C) > 5.0%	60.1%	65.3%	8	C/C
Rayis 2020	[39]	P. COH: 68 GDM, 272 NGT	IADPSG	Glycated haemoglobin(HbA1C) > A = 4.1%, B = 5.8%	A = 76.5% B = 13.4%	A = 37.8% B = 91.4%	8	A = C/D B = D/A
Ghosh 2017	[33]	CC: 500 NGT, 127 GDM	C&C	Glycated CD59 = cut-off not specified	85%	92%	10	B/A: APN
Cakmak 2019	[103]	CC: 60 GDM, 75 NGT	C&C	Vascular adhesion protein 1 (VAP-1) > 2.3	70%	65.3%	8	C/C
Tekin 2020	[127]	CC: 30 NGT, 50 GDM	IADPSG	Signal peptide-CUB-EGF domain-containing protein (SCUBE)-1 > 36.8 ng/mL	93.1%	74.2%	10	A/C
Dudzik 2015	[42]	CC: 20 GDM, 30 NGT	WHO 5.6,8.9,7.8	LPE(20:1), (20:2), (22:4); LPC(18:2), (20:4), (20:5); LPI(18:2), (20:4); LPS(20:0) and LPA(18:2)	All 100%	All 95%	9	A/A: LCPSN
Sanchez-Garcia 2020	[128]	P.COH: 38 GDM, 102 NGT	IADPSG	Triglyceride-glucose index (TyG) > 4.69 ng/mL	89%	50%	9	B/D
Gingras 2018	[129]	P.COH: 73 GDM, 1415 NGT	C&C	Fructosamine > 222 _mol/L	55%	49%	9	C/C
Koroglu 2019	[90]	CC: 50 GDM, 30 NGT	IADPSG	Pancreatic-derived factor (PANDER) > 227.2 ng/ml	100%	87%	9	A/B: LCPSN
Pan 2019	[63]	COH: 96 GDM,304 NGT	IADPSG	Betatrophin > 106 pg/mL	69%	84%	9	C/C
Fatima 2017	[20]	CC: 208 GDM, 300 NGT	IADPSG	A = CHEMERIN > 415.49 ng/ml B = LEPTIN > 48.5 ng/ml	A = 96% B = 90%	A = 72% B = 96%	9	A = A/C B = A/A: APN
Yuan 2018	[29]	COH: 87 GDM, 273 NGT	IADPSG	Ficolin-3/adiponectin ≥ 1.06	91%	97%	11	A/A: APN
Ning 2016	[83]	CC: 46 GDM,55 NGT	IADPSG	FABP4 > 1.96 ng/mL	89%	87%	10	B/B: APN
Wang 2020	[130]	CC: 60 GDM, 50 NGT	IADPSG	Fatty acid-binding protein 4 (FABP4) > 27.64 Chemerin > 6.78 Chemerin + FABP > 0.71	75% 73.3% 80%	80% 76% 96%	10	C/B B/B B/A: APN
Tawfeek 2017	[68]	CC: 45 GDM, 45 NGT	IADPSG	Sex hormone binding globulin (SHBG) > 50 nmol/L	90%	96%	10	A/A: LCPSN
Amirian 2019	[66]	P. COH: 63 GDM, 460 NGT	IADPSG	Unconjugated Estriol (UE) > 0.965 MOM	66.6%	54.8%	11	C/C
Yilmaz 2014	[131]	CC: 42 GDM, 68 NGT	C&C	Neutrophil to Lymphocyte Ratio (NLR) > 2.93	76%	94%	**9**	C/B
Butt 2017	[132]	CC: 59 GDM, 41 NGT	IADPSG	Cobalamin > 113 pg/mL	57%	80%	10	C/B
Giacobbe 2016	[133]	CC: 75 GDM,48 NGT	IADPSG	High mobility group box 1 > 1.85 ng/ml	81%	96%	11	B/A:APN
Shaas 2017	[82]	CC: 50 GDM,50 NGT	IADPSG	risk factors + HBA1c + SHBG + PAPP-A + CRP	75%	91%	10	C/A
Thériault 2016	[81]	CC: 264 GDM,528 NGT	IADPSG	previous GDM, family history of diabetes and soft drink intake before pregnancy + HbA1c + SHBG + BMI	69%	90%	12	C/A
Tantanasis 2010	[14]	CC: 20 GDM, 15 NGT	WHO 99	Fetus maximum subcutaneous fat tissue thickness > 3.950 mm at HC, 4.550 mm at AC and 4.700 mm at TS	100%	100%	11	A/A: LCPSN
Kansu-Celick 2018	[108]	COH: 46 GDM,177 NGT	C&C	Maternal Subcutaneous adipose tissue (SAT) thickness > 16.75 mm	72%,	58%	9	C/C
Aydin 2020	[115]	CC: 60 GDM, 60 NGT	C&C /IADPSG	Fetal epicardial fat thickness (fEFT) > 0.95 mm	65%	88%	8	C/B
İlhan 2018	[111]	CC: 33 GDM, 64 NGT	IADPSG	Fetal liver volume (FLV) > 32.72 cm3	79%	56%	9	C/C
Perovic 2012	[117]	COH: 33 GDM, 77 NGT	C&C	Ultrasound Gestational Diabetes Screening Score (UGDS)^c^ > 4	B = 91%,	B = 90%	11	A/A: APN

^a^A = very good sensitivity/specificity (> 90%), B = good sensitivity/specificity (80–89%), C = low sensitivity/specificity (70–79%), D = very low sensitivity/specificity (< 70%); LCPSN = Larger Challenge Phase Study Needed if population < 100

^b^UGDS score = Increased adipose subcutaneous tissue, cardiac width, cardiac circumference, placental thickness, Polyhydramnion, Asymmetrical macrosomia, Thickened intra-ventricular septum, Intensified breathing movements, Immature appearance of placenta

##### OGTT avoidability

Four articles reported the number of OGTT potentially avoidable by using the biomarker described in their studies as the screening test (Table 4). Two articles assessed fasting capillary/plasma glucose [15, 31] and two glycated haemoglobin [34, 36]. The number of OGTT avoidable was calculated as a sum of the number of patients having values below the screening and above the diagnostic thresholds for each biomarker. These thresholds were identified with ROC analysis: the diagnostic threshold was set with specificity between 100% [15, 31, 36] and 97.2% [34], with the screening threshold sensitivity between 26.4% [36]  and 96.9% [15]. The avoidable OGTT ranged from 38% [36]  to 61.8% [34] using Hba1c > 5.8% or 5.4% and from 61.3 to 81.3% with FBG > 4.4– 4.7 mmol [15] 9.9% respectively.

**Table 4:** Biomarkers assessed as screening tool (number of OGTT avoidable)

Author—year	Ref N	Biomarker	Diagnostic criteria	Screening threshold value (sensitivity %)	Diagnostic cut-off value (specificity %)	OGTT avoided
Trujillo 2014	[15]	Fasting blood glucose (FBG)	IADPSG	A = 4.4 mmol⁄ l (96.9%) B = 4.7 mmol⁄ l (92.5%)	5.1 mmol⁄ l (100%)	A = 61.3% B = 81.3%
Ruetschi 2016	[31]	Fasting blood glucose (FBG)	IADPSG	4.4 mmol⁄ l (78.5%)	5.1 mmol/l (100%)	63.8%
Renz 2015	[36]	Glycated haemoglobin (HbA1C)	WHO 1999	5.8% (26.4%)	6.5% (100%)	B = 38%
Rajput 2012	[34]	Glycated haemoglobin (HbA1C)	ADA/IADPSG	5.45% (85.7%)	5.95% (97.2%)	61.8%

### Discussion

#### Summary of evidence

We identified a diverse range of biomarkers differing between GDM and NGT pregnancies, in maternal/cord blood, amniotic fluid and placental samples, as well as at ultrasound examination. Many of the included studies, though, despite reporting statistical significance, only found very small absolute differences between GDM and control, reducing the potential clinical utility of these biomarkers as stand-alone diagnostic markers. There were few biomarkers that differed to a statistically significant and clinically meaningful extent between GDM and NGT. Further research to explore their potential utility as replacements for the OGTT, whether alone or in combination with other biomarkers, is warranted.

The most common biomarkers evaluated were haematological. Among these, HbA1c and FBG were assessed in the largest sample sizes and for their ability to avoid OGTT; though neither have been shown to fully substitute for OGTT. A previous systematic review on the use of HbA1c for the diagnosis of GDM found overall high specificity but low sensitivity concluding that “HbA1c should only be used in association with other standard diagnostic tests for GDM diagnosis”[134].

Among the almost 150 biomarkers evaluated in Challenge-phase studies, Leptin [20], Ficolin3/adiponectin ratio [52] and Chemerin/FABP [135] had promising results, yielding very good sensitivity and specificity (> 90%) in adequate sample sizes (= / > 100). Haematological biomarkers demonstrating very good sensitivity and specificity, needing to be confirmed in larger cohorts of at least 100 patients to assess potential substitution for OGTT, are Sex hormone binding globulin (SHBG) and metabolomic profiling for phospholipids, though the latter may be too expensive to be used as a screening or diagnostic test for GDM. Finally, challenge studies are needed to test the sensitivity/ specificity of all the haematological biomarkers reported to be significantly different in GDM. Among those, Adiponectin, AFABP, Betatrophin, CRP, Cystatin-C, Delta-Neutrophil Index, GGT, TNF-A were those demonstrating statistically and potentially clinically significant differences in substantial cohorts of patients (> 500).

Amniotic fluid biomarkers clearly have a limited utility as they are only justifiable in those otherwise requiring interventional sampling. Among the several ultrasound features described as differing in GDM, fetal subcutaneous fat thickness (FSFT) and the UGDS score have been assessed in challenge-phase studies demonstrating promising results. FSFT needs to be confirmed in a cohort of at least 100 women [13], whereas UGDS score can be evaluated in Advanced-phase studies [117]. Post-partum analysis of fetal blood analytes confirmed the higher adipogenic environment found in GDM women as well as the hormonal imbalance in terms of insulin resistance, though none of these biomarkers was investigated in a large cohort and clearly have no prospective utility. The same can be said for placental histomorphological alterations, though either of these markers could potentially be used to correlate between OGTT, alternative screening tests and eventual outcome with regard ‘true’ GDM in advanced-phase studies.

Whilst several biomarkers show differentiation between GDM and NGT pregnancies, practicalities and translatability need to be taken into account along with sensitivity and specificity, as recommended by WHO ASSURED criteria: a biomarker should be affordable, sensitive, specific, user friendly, rapid and robust, equipment-free and deliverable to end-users [136]. None of the two biomarkers assessed as a potential screening test, namely FBG and HbA1c, could fully replace OGTT. Furthermore, certain haematological biomarkers (especially metabolomics) could be too expensive, time-consuming or require invasive amniotic fluid assessment. Ultrasound markers could represent a good trade-off between cost and acceptability/feasibility, provided the assessment technique is easy and standardisable.

The lack of a gold standard to confidently identify GDM represents a limitation to any study assessing a new diagnostic tool, as most of the new tools are judged against the existing imperfect screening test (the OGTT). There is also a lack of consensus for GDM diagnostic criteria, with some articles authored in 2019–2020 still using NDDG criteria from 41 years previously [137, 138]. Screening failures with the OGTT are well documented along with the potential for false positive and false negative results [139, 140]. Data triangulation could represent a solution to this limitation, a process described as “the application of (at least) two different methods aimed at one particular problem” [141]. The results of OGTT could be combined with those of an alternative diagnostic method as well as with risk factors and outcomes of GDM, potentially including post-natal biomarkers such as placenta histomorphology.

Whilst many biomarkers presented in this review are not suitable as stand-alone markers, they could potentially be included in a multi-modal/triangulated evaluation of OGTT positive and negative patients. This advanced-phase analysis might allow a new and more complete understanding of detection and definition of GDM. As per Hackfort et al. [142], data triangulation is “relating different data or sources of data in such a way that will result in a new picture of the object, a different construction of the object, and a new idea of the object”. Chikere and Wilson recently reviewed diagnostic test evaluation methodology in the absence of gold standard with multiple imperfect reference standards used, identifying Discrepancy Analysis (DA) and Latent Class Analysis (LCA) to be the most suitable methodology [143]. DA “compares the index test with an imperfect reference standard: participants with discordant results undergo another imperfect test, called the resolver test, to ascertain their disease status”. “To avoid biased estimates, some of the participants with concordant responses (true positives and true negatives) can be sampled to undertake the resolver test alongside participants with discordant responses (false negative–FN and false positive–FP)”. In comparison, using LCA, the test performance of all the tests employed in the study are evaluated simultaneously using probabilistic models with the basic assumption that the disease status is latent (frequentist LCAs) or unobserved (Bayesian LCAs). DA and LCA could represent the best way to triangulate the most promising biomarkers of GDM in advanced-phase studies.

#### Strengths and limitations

To the best of our knowledge, this is the first systematic review on GDM biomarkers, reporting the results of 174 articles for almost 136,000 participants, reviewed according to PRISMA checklist and assessed using CASP criteria for quality of included publications, setting up a CASP checklist threshold to exclude studies not fulfilling multiple criteria thereby reducing the risk of bias. Incomplete retrieval of identified research was minimised by screening the references of 20% of the included articles; identifying 22 additional articles.

The heterogeneity of methods used in the included articles to diagnose GDM and to assess the biomarkers is a limitation that precluded meta-analysis and allowed only narrative synthesis. The preponderance of studies from just two countries with specific ethnic backgrounds (China and Turkey) may limit the external validity of the findings.

## Conclusions

Whilst multiple biomarkers may show differences between GDM and non-GDM pregnancies, few of these differences were of sufficient absolute size or of a nature to be clinically useful. The most promising biomarkers for detection of GDM were: Leptin, Ficolin – 3/Adiponectin and Chemerin/FABP among the haematological biomarkers and UGDS score at ultrasound examination. No single feature currently performs sufficiently well to be an adequate screening test for GDM. We hope that this current work will provide a guide for future research evaluating indicators for GDM in challenge-phase studies and advanced-phase studies on larger and more diverse populations, assessing predictive value, and patient acceptability. Triangulation of these biomarkers values, alone or in combination, with the results of OGTT and GDM risk factors and outcomes, may potentially lead to more efficacious screening tools for GDM than the current OGTT.

## Supplementary Information

**Additional file 1.** **A** Characteristics of included studies – exploratory phase of haematological biomarkers: Challenge Phase Available (CPA), Challenge Phase Needed (CPN) and Not Significant Results (NSR). **B** Characteristics of included studies – exploratory phase of amniotic fluid biomarkers: Challenge Phase Available (CPA), Challenge Phase Needed (CPN) and Not Significant Results (NSR). **C** Characteristics of included studies – exploratory phase of ultrasound biomarkers: Challenge Phase Available (CPA), Challenge Phase Needed (CPN) and Not Significant Results (NSR). **D** Characteristics of included studies – exploratory phase of fetal/annexes’ biomarkers: Challenge Phase Available (CPA), Challenge Phase Needed (CPN) and Not Significant Results (NSR). Table summarising the results of individual studies separated into the different types of biomarkers

**Additional file 2**. Table reporting the PRISMA GUIDELINE CHECKLIST

**Additional file 3**. Table used to extract data from the articles assessed

**Additional file 4**. Table of all the biomarkers evaluated divided into category and described with their name, abbreviation, function.

## Data Availability

The data sets reside with the authors of the original papers evaluated.

## Abbreviations

ACHOIS: Australian Carbohydrate Intolerance Study in Pregnant Women
ADA: American Diabetes Association
BGL: Blood Glucose Level
CASP: Critical Appraisals Skill Programme
C&C: Carpenter and Coustan
GCT: Glucose Challenge Test
GDM: Gestational Diabetes Mellitus
HAPO: Hyperglycemia and Adverse Pregnancy Outcome
IADPSG: International Association of Diabetes in Pregnancy Study Groups
NDDG: National Diabetes Data Group
NGT: Normal Glucose Tolerance
OGTT: Oral Glucose Tolerance Test
WHO: World Health Organization
