# Supplementary material for: The diagnostic indicators of gestational diabetes mellitus from second trimester to birth: a systematic review
Source: Clin Diabetes Endocrinol. 2021 Oct 11;7:19. doi: 10.1186/s40842-021-00126-7 (PMC8504031; doi:10.1186/s40842-021-00126-7)
Supplement: Supplementary file 1 — Additional file 1. A Characteristics of included studies – exploratory phase of haematological biomarkers: Challenge Phase Available (CPA), Challenge Phase Needed (CPN) and Not Significant Results (NSR). B Characteristics of included studies – exploratory phase of amniotic fluid biomarkers: Challenge Phase Available (CPA), Challenge Phase Needed (CPN) and Not Significant Results (NSR). C Characteristics of included studies – exploratory phase of ultrasound biomarkers: Challenge Phase Available (CPA), Challenge Phase Needed (CPN) and Not Significant Results (NSR). D Characteristics of included studies – exploratory phase of fetal/annexes’ biomarkers: Challenge Phase Available (CPA), Challenge Phase Needed (CPN) and Not Significant Results (NSR). Table summarising the results of individual studies separated into the different types of biomarkers [file 40842_2021_126_MOESM1_ESM.docx]

Additional file 1.a Characteristics of included studies – exploratory phase of haematological biomarkers: Challenge Phase Available (CPA), Challenge Phase Needed (CPN) and Not Significant Results (NSR)

| **AUTHOR, YEAR** | **REFERENCE N** | **DESIGN: SAMPLE SIZE** | **GDM DIAGNOSTIC CRITERIA** | **GA** | **BIOMARKER** | **GDM vs NGT > / = / <** | **VALUES (P<0.05)** | **COMMENTS** |
| --- | --- | --- | --- | --- | --- | --- | --- | --- |
| **VARIATIONS OF OGTT/ GLYCATION END PRODUCTS/ FULL BLOOD COUNT FEATURES** | | | | | | | | |
| *Rüetschi, 2016* | [1] | PROSP COH: 2047 NGT, 251 GDM | IADPSG | 24-28 | Fasting blood glucose (FBG) | > | ≥92 mg/dL in 5% of total cohort and 47% of GDM | CPA |
| *Trujillo, 2014* | [2] | RETRO COH: 4040 NGT,886 GDM | IADPSG | 24-28 | Fasting plasma glucose (FPG) | > | >92 mg/dl in 767 of the 888 GDM | CPA |
| *D’emden 2020* | [3] | RETRO COH: 3946 GDM, 22296 NGT | IADPSG | 24-32 | Fasting plasma glucose (FPG) | > | > 91.8 mg/dl in 8.6% of total cohort | CPA |
| *Anjalakshi, 2009* | [4] | PROSP COH: 713 NGT, 87 GDM | WHO 99 | 16-32 | BGL at 2 hours after 75g glucose | > | * | CPA |
| *Ghosh, 2017* | [5] | CC: 500 NGT, 127 GDM | C&C | 24-28 | Plasma glycated CD59 (pGCD59) | > | 3.23 vs 0.33 SPU | CPA |
| *Rajput, 2012* | [6] | PROSP COH: 43 GDM,560 NGT | C&C -IADPSG | 24-28 | Glycated haemoglobin (HbA1c) | > | 39 vs 34 mmol/L | CPA |
| *Kwon, 2015* | [7] | RETRO COH: 242 GDM, 79 NGT | C&C | 23-30 | Glycated haemoglobin (HbA1c) | > | 37 vs 31 mmol/L | CPA |
| *Renz, 2015* | [8] | CC: 176 NGT, 86 GDM | C&C - IADPSG | 22-32 | Glycated haemoglobin (HbA1c) | > | 37 vs 32 mmol/mol | CPA |
| *Siricharoenthai 2020* | [9] | CC: 35 GDM , 79 NGT | NDDG | 23-35 | Glycated haemoglobin (HbA1c) | > | 34.4 vs.30.1 mmol/mol | CPA |
| *Khan 2020* | [10] | PROSP COH: 50 GDM,230 NGT | IADPSG | 15-29 | Glycated haemoglobin (HbA1c) | > | 43.2 vs 35.5 mmol/mol | CPA |
| *Lai 2020* | [11] | RETRO COH: 3547 GDM, 15714 NGT | IADPSG | 24-28 | Glycated haemoglobin (HbA1c) | > | 31.6 *vs. 29.2* mmol/mol | CPA |
| *Rays 2020* | [12] | PROSP COH: 68 GDM, 272 NGT | IADPSG | 24-30 | Glycated haemoglobin (HbA1c) | > | 29 VS 26.8 mmol/mol | CPA |
| *Huang, 2015* | [13] | PROSP COH: 661 NGT, 232 GDM | IADPSG | 20-36 | Glycated albumin (GA) | > | * | CPN |
| *Zhu, 2017* | [14] | PROSP COH: 232 GDM, 466 NGT | IADPSG | 24-28 | HbA1c GA FPG | All > | 5.2 vs 5.0% 12.1 vs 11.8% 4.9 vs 4.6 mmol/L | CPA |
| *Li, 2019* | [15] | CC: 90 GDM, 90 NGT | IADPSG | 24-29 33-41 | Advanced Glycation End-products (Ages) | > | 473.6 vs 324.4 ng/L & 533.5 vs 315.5 ng/L | CPN |
| *Bartakova, 2016* | [16] | CC: 222 GDM , 85 NGT | WHO 99 | 24-30 | Nε- (carboxymethyl)lysine (CML) | > | 683.6 vs 507.5 ng/mL | CPN |
| *Gingras, 2018* | [17] | PROSP COH: 73 GDM, 1415 NGT | C&C | 24-28 | Plasma Fructosamine | > | 11.6 mmol/L | CPA |
| *Çeltik, 2016* | [18] | CC: 105 GDM, 40 NGT | C&C | 24-48 | Mean platelet volume (MPV) | > | 8.7 vs 8.3 fL | CPN |
| *Sargin, 2016* | [19] | RETRO COH: 144 GDM ( 76 IGT) NGT 304 | C&C | 24-30 | Neutrophil-to-lymphocyte ratio (NLR) Platelet-to-lymphocyte ratio (PLR) | = | Not significant difference | NSR |
| *Yilmaz, 2013* | [20] | CC: 42 GDM, 68 NGT | C&C | 24-28 | NLR | > | 3.0 vs. 2.3 | CPA |
| *Aktulay, 2016* | [21] | CC: 29 GDM, 29 NGT | C&C | 34-40 | Chitinase-3-like protein 1 (YKL-40), NLR PLR | All > | 203 *vs* 159.2 ng/mL 5.1 *vs* 3.3 144.7 vs 114.4 | CPN |
| **METABOLISM MARKERS, LIPIDS, ADIPOSE TISSUE MARKERS AND ADIPOKINES** | | | | | | | | |
| *Yuan, 2018* | [22] | PROSP COH: 86 GDM, 273 NGT | IADPSG | 16-18 24-28 37-41 | Secreted frizzled-related protein 4 (SFRP4), Ficolin-3 | All > | 12.8 *vs* 10.2 ng/mL, . 10.6 vs 8.2 lg/mL | CPN |
| *Dudzick, 2014* | [23] | CC: 20 GDM, 30 NGT | WHO 5.6,8.9,7.8 | 22-28 | Metabolomic profiling – Lysophospholipids, | > | * | CPA |
| *Chen, 2018* | [24] | CC: 49 GDM, 44 NGT | IADPSG | 26-28 | Metabolomic profiling - 2-aminobutyric acid | > | * | CPN |
| *Zheng, 2017* | [25] | CC: 30 NGT, 30 GDM | IADPSG | 20 | Metabolomic profiling - linolenic acid and arachidonic acid | > | * | CPN |
| *De seymour, 2014* | [26] | CC: 22 GDM, 26 NGT | EASD/ADIPS 75g OGTT | 20 | Metabolomic profiling - Itaconic acid | > | * | CPN |
| *Zhao, 2015* | [27] | PROSP COH: 24 GDM ,116 NGT | IADPSG | 24-28 32-35 | Long-chain polyunsaturated fatty acids (LCPUFAs) | > | * | CPN |
| *Yi, 2017* | [28] | PROSP COH: 141 GDM, 230 NGT | IADPSG | 24-28 | 3-carboxy-4-methyl-5-propyl-2-furanpropanoic acid (CMPF) | ? | 92.9 vs. 81.9. μM | CPN |
| *Whang, 2015* | [29] | CC: 110 GDM, 526 NGT | IADPSG | 24-28 | Triglyceride to high-density lipoprotein cholesterol (TG/HDL-C) ratios | > | 1.24 vs 1.04 | CPN |
| **AUTHOR, YEAR** | **REFERENCE N** | **DESIGN SAMPLE SIZE** | **GDM DIAGNOSTIC CRITERIA** | **GA** | **BIOMARKER** | **GDM vs NGT > / = / <** | **VALUES (P<0.05)** | **COMMENTS** |
| *Sanchez-Garcia 2020* | [30] | PROSP COH: 38 GDM, 102 NGT | IADPSG | 24-28 | Triglyceride-glucose index (TyG) | > | 4.88 vs 4.68 | CPA |
| *Alyas 2019* | [31] | CC: 58 GDM, 100 NGT | IADPSG | 14-18, 24-28 | Triglycerides Total cholesterol (TC) Low density lipids (LDL) Very low-density lipids (VLDL) High density lipids (HDL) Hscrp | >/>/>/>/</> | 369.5 vs 346.4 mg/dL & 450.4 vs 423.2 308.9 vs 287.7 mg/dL & 367.9 vs 340.8 165.6 vs 131.2 mg/dL & 227.1 vs 201.6 mg/dL 39.9 vs 31.9 mg/dL & 54.4 vs 47.4 mg/dL 45.7 vs 59.8 mg/dL & 33.4 vs 41.4 mg/dL 6.2 vs 4.7 mg/L & 9.9 vs 7.3 mg/L | CPN |
| *Retnakaran, 2019* | [32] | PROSP COH: 214 GDM, 629 NGT | NDDG | 27-33 | Apolipoprotein A1 (ApoA1) | = | No significant difference | NSR |
| *Ghaneei, 2015* | [33] | CC: 46 GDM, 46 NGT | IADPSG | 24-26 | Oxidised low-density lipoprotein (OxLDL) | > | 17.2 vs. 8.8 U/L | CPN |
| *Yuan, 2018* | [34] | PROSP COH: 86 GDM, 273 NGT (same cohort of WOS 101) | IADPSG | 16-18 | Ficolin-3, Ficolin-3/adiponectin ratio Adiponectin | > > < | 10.6 vs 8.2 lg/mL 1.3 vs 0.9 8.6 vs 10.7 lg/mL. | CPA |
| *Bagci, 2017* | [35] | CC: 40 GDM, 40 NGT | NDDG | 24-28 | Adiponectin Resistin | </> | * | CPN |
| *Karasek 2020* | [36] | CC: 35 GDM, 50 NGT, (50T2DM) | IADPSG | 26-30 | Adiponectin AFABP | </= | 9.1 vs 11.6 μg/ml No significant difference | CPN |
| *Şengül, 2009* | [37] | CC: 20 GDM (20 IGT) 20 NGT | C&C | 24-32 | Leptin | > | No significant difference | NSR |
| *Fatima, 2017* | [38] | CC: 208 GDM, 300 NGT | IADPSG | Late second trimester | Chemerin, Interleukin-18 (IL-18) Leptin | > | 93.4 vs 14.3 ng/mL 161 vs 39 ng/L 20.4 vs 3.4 ng/mL | CPA |
| *Ortega-senovilla, 2011* | [39] | CC: 98 GDM, 86 NGT | C&C | 38-39 | Adipocyte fatty acid-binding protein (AFABP) Retinol-binding protein 4 (RBP4) Adiponectin | >/>/< | 20 vs 18 ng/mL 124 vs 60 ng/mL 6 vs 8 ng/mL | CPN |
| *Kralisch, 2009* | [40] | CC: 40 GDM, 80 NGT | WHO 99 | 29-35 | AFABP | > | 22.9 vs 18.3mg/l | CPN |
| *Wong 2020* | [41] | CC: 60 GDM, 50 NGT | IADPSG | 22-27 | Fatty acid‑binding protein 4 (FABP4) Chemerin | > | 35.1 vs 21.5 μg/l 7.7 vs 5.8 μg/l | CPA |
| *Liu 2020* | [42] | CC: 61 GDM , 122 NGT | IADPSG | 23-27 | Coiled-coil domain-containing 80 (CCDC80) | < | 0.2 vs 0.3 pg/mL | CPN |
| *Ning, 2016* | [43] | CC: 46 GDM, 55 NGT | IADPSG | 22-30 | Fatty acid-binding protein 4 (FABP4) TNF-a | > | 4.8 vs. 1.1 ng/mL 90.2 vs. 18.6 pg/mL | CPN |
| *Zhang, 2016* | [44] | PROSP COH: 40 GDM, 240 NGT | IADPSG | 24-28 , 37-41 | AFABP Leptin RBP4 Adiponectin | >/< | 32.3 vs 22.0 µg/L & 36.5 vs 21.8 µg/L 10.6 vs 4.7 µg/L & 13.7 vs 6.3 µg/L 49.3 vs 36.8 mg/L & 51.8 vs 39.3mg/L 4.0 vs 7.3 mg/L & 3.3 vs 7.4 mg/L | CPN |
| *Su 2010,* | [45] | CC: 63 GDM, 58 NGT | C&C | 24-28, | Retinol-binding protein 4 (RBP4) | > | 41.6 vs 34.5 mg/L | CPN |
| **HORMONES AND THEIR TRANSPORT MOLECULES, GROWTH FACTORS, NEUROPEPTIDES, VITAMINS, AND IRON STUDY** | | | | | | | | |
| *Tawfeek 2017* | [46] | CC: 45 GDM, 45 NGT | IADPSG | 24-28 | Sex hormone binding globulin (SHBG) | < | 23 vs 78 nmol/L | CPA |
| *Pan 2019* | [47] | PROSP COH: 96 GDM, 304 NGT | IADPSG | 24-28 | Betatrophin | > | 589 vs 343 pg/mL | CPA |
| *Ebert 2015* | [48] | CC: 74 GDM. 74 NGT | IADPSG | 22-34 | Betatrophin | > | 1.8 vs 1.6 mg/L | CPN |
| *Erol 2015,* | [49] | CC: 45 GDM, 45 NGT | IADPSG | 24-28 | Betatrophin | > | 635.8 vs 320.1 ng/L | CPN |
| *Zhong 2020* | [50] | CC: 40 GDM, 40 NGT | IADPSG | 18- 20, 24-28 | Aprosin | > | 1.3 vs 0.5 & 0.6 vs 0.5 ng/mL | CPN |
| *Gorar 2017* | [51] | CC: 110 GDM, 159 NGT | C&C | 24-28 | Thyroid Stimulating Hormone (TSH) Free T3 Free T4 | >/</< | 1.3 vs 1.0 μIU/mL 10.4 vs 10.6 fL 2.9 vs 3.1 pg/mL | CPN |
| *Amirian 2019* | [52] | PROSP COH: 63 GDM, 460 NGT | IADPSG | 14-17 | Unconjugated Estriol (UE) | > | 1.15 vs 1.05 multiples of the median (MOM) | CPA |
| *Liu 2020* | [53] | CC: 50 GDM, 47 NGT | IADPSG | 37-42 | Transthyretin (TTR) RBP4/TTR ratio | >/< | 93.4 vs. 80.8 μg/ml 517.6 vs. 602.6 | CPN |
| *Ebert 2016* | [54] | CC: 74 GDM, 74 NGT | IADPSG | 22-34 | Copeptin | < | 3.5 vs 4.4 pmol/l | CPN |
| *Zhang 2015* | [55] | CC: 25 NGT, 25 GDM | IADPSG | 24-28 | Galanin SHBG | >/< | 26.0 vs. 19.1 pg/Ml 25.9 vs. 45.7 nmol/L | CPN |
| *Zhang 2014* | [56] | CC: 30 GDM , 30 NGT | IADPSG | 24-28 | Galanin GGT | > | * | CPN |
| *Nergiz 2014* | [57] | CC: 20 GDM, 30 NGT | C&C | 26-36 | Galanin IL-6 | > | 44.9 vs 15.7 pg/mL 32.5 vs 7.1 pg/mL | CPN |
| **AUTHOR, YEAR** | **REFERENCE N** | **DESIGN SAMPLE SIZE** | **GDM DIAGNOSTIC CRITERIA** | **GA** | **BIOMARKER** | **GDM vs NGT > / = / <** | **VALUES (P<0.05)** | **COMMENTS** |
| *Nӓf, 2014* | [58] | PROSP COH: 129 NGT, 78 GDM | NDDG | 26-30 | Follistatin Follistatin-like-3 (FSTL3) Activin A | </=/= | 8,216.3 vs 9,2263.4 ng/mL | CPN |
| *Yuksel 2015* | [59] | CC: 35 NGT, 45 GDM | C&C | 24-28 | Atrial natriuretic peptide (ANP) Brain-type natriuretic peptide (BNP) l | < | 12.9 vs 34.8 pg/ml 416.6 vs 629.7 mg/dl | CPN |
| *Kucukler 2016* | [60] | CC: 38 GDM, 41 NGT | C&C | 15-31 | Nesfatin-1 Insulin | </> | 755.0 vs 832.3 ng/ml 16.2 vs 10.5 mIU/ml | CPN |
| *Ademoglu 2016* | [61] | CC: 40 GDM, 30 NGT | C&C | 24-28 | Nesfatin-1 | < | 7.9 vs 11.2 ng/mL | CPN |
| *Mierzynski 2019* | [62] | CC: 153 GDM, 84 NGT | IADPSG | 25-29 | Nesfatin-1 Vaspin | < | 5.1 vs 6.7 ng/mL 1.3 vs 1.7 ng/mL | CPN |
| *Yavukzir 2020* | [63] | CC: 30 GDM, 30 NGT | IADPSG | 24-38 | Subfatin Spexin | > | * | CPN |
| *Kuruk Eken 2017* | [64] | CC: 63 GDM, 64 NGT | IADPSG | 24-28 | Neuregulin 4 | > | 3.6 vs 1.8 ng/mL | CPN |
| *Butt 2017* | [65] | CC: 59 GDM, 41 NGT | IADPSG | 34-42 | Cobalamin | < | 108.95 vs 173 pg/mL | CPA |
| *Khosrowbeygi 2020* | [66] | CC: 49 GDM, 51 NGT | IADPSG | 24-28 | 25-hydroxyviatmin D (25 OHD) | < | 13.0 vs 17.6 ng/mL | CPN |
| *Yaqiong 2020* | [67] | CC: 110 GDM, 100 NGT | IADPSG | >24 | 25-hydroxyviatmin D (25 OHD) | < | 13.9 vs 17.5 ng/mL | CPN |
| *Sarker 2011* | [68] | PROSP COH: 43 NGT, 57 GDM | WHO 1997 Birdem | 21-36 | HbA1c Insulin Hb Iron | >/>/=/< | 6.09 vs 5.8% 2.5 vs 1.5 ng/mL No significant difference 6 vs 12 | CPN |
| *Afkhami-ardekani 2009* | [69] | CC: 34 NGT,34 GDM | C&C | 24-28 | Iron Hb MCV MCH Transferrin saturation Ferritin TIBC | >/>/>/>/>/< | Iron 100.4 vs 56.8 13.4 vs 11.7 g/dl 85.3 vs 77.7 fl 46.9 vs 22.1 pg 26.5 vs 12.8 73.3 vs 41.5 ng/ml 383.1 vs 457.8 μg/dl | CPN |
| *Alam 2017* | [70] | CC: 44 GDM, 44 NGT | IADPSG | 28-36 | Serum transferrin receptor protein (sTfR) Ferritin Hs-CRP | = | Not significant difference | NSR |
| **OXIDATIVE STRESS, ANTIOXIDANTS, INFLAMMATION, AND IMMUNE SYSTEM MARKERS** | | | | | | | | |
| *Rueangdetnarong 2018* | [71] | CC: 30 GDM, 32 NGT | NDDG | 24-28/Early Labour | 8 Isoprostane (8Isop) Tumour necrosis factor alpha (TNF-α) Interluekin 10 (Il-10) | >/>/= | 737.5 vs 249.1 & 666.4 vs 104.8 4.70 vs 1.75 Not significant difference | CPN |
| *Li 2016* | [72] | CC: 22 GDM, 30 NGT | IADPSG | 16-20/ 24-28 /32-36 | 8-iso-prostaglandin (8-iso-PGF2𝛼) Advanced oxidative protein products (AOPP) Protein Carbonyl (PCO) Glutathione Peroxidase-3 (GPX-3) Paraoxonase-1 (PON1) | >/>/>/>/< only (16-20 and 32-36) | 196.1 vs 154.8 ng/mL & 538.7 vs 381.1 ng/mL 64.7 vs 60.0 𝜇mol/L & 83.6 vs 72.9 𝜇mol/L . 172.8 vs 136.8 ng/mL & 232.5 vs 180.81 ng/mL 160.4 vs 83.7 𝜇IU/mL & 35.6 vs 26.5 𝜇IU/mL 162.9 vs 260.2 mIU/mL | CPN |
| *Gelisgen 2011* | [73] | CC: 23 GDM , 22 NGT | C&C | 24-28 | Protein carbonyl (PCO) Protein hydroperoxides (P-OOH) Advanced oxidation protein products (AOPP) Nitrotyrosine (NT) Paraoxonase (PON-1) | >/>/>/>/< | 1.3 vs 0.9 nmol/mg 1.5 vs 0.9 ng/mL 53.3 vs 43.8 ng/mL 0.7 vs 0.3 nM 95.3 vs 133.1 U/mL | CPN |
| *Lewandowski 2014* | [74] | PROSP COH: 20 NGT (15 IGT) 16 GDM | WHO 99 | 28 | Lipid peroxidation products (LPO) TNF-a soluble receptors (sTNF-R1 and sTNF-R2) Soluble adhesion molecules (sICAM-1, sVCAM-1) | >/=/= | 64.1 vs 47.0 ng/mL not significant difference not significant difference | CPN |
| *Beyazit 2020* | [75] | CC: 45 GDM, 45 NGT | IADPSG | 24-38 | ischemia modified albumin (IMA) total oxidative stress (TOS) oxidative stress index (OSI) betatrophin | =/>/>/= | not significant difference 11.7 vs 7.2 21.8 vs 11.9 not significant difference | CPN |
| *Ma 2012* | [76] | CC: 40 GDM / 30 NGT | C&C | 26-29/30-34 | Ischemia-modified albumin (IMA) | > | 86.4 vs 65.8 U/L & 75.8 vs 68.1 U/L | CPN |
| *Ozler 2019* | [77] | CC: 57 GDM, 29 NGT | C&C | 24-28 | A Disintegrin and Metalloproteinase with thrombospondin motifs 5 (ADAMTS5), Total antioxidant status (TAS) Total oxidant status (TOS) Oxidative stress index (OSI) | </=/>/> | 13.9 vs 18.8 pg/mg < (values above) Not significant difference 9.2 vs 5.5 nmol 2HO Eq/mg 5.1 vs 3.4 arbitrary unit (AU) | CPN |
| **AUTHOR, YEAR** | **REFERENCE N** | **DESIGN SAMPLE SIZE** | **GDM DIAGNOSTIC CRITERIA** | **GA** | **BIOMARKER** | **GDM vs NGT > / = / <** | **VALUES (P<0.05)** | **COMMENTS** |
| *Parast 2017* | [78] | CC: 40 GDM, 40 NGT | C&C | 24 – 28 | Total antioxidant capacity (TAC) | < | 2.3 vs. 3.7 μmol/L | CPN |
| *Cakina 2020* | [79] | CC: 45 GDM, 45 NGT | IADPSG | 22-34 | Disulfide, disulfide/total thiol, disuplhide/native thiol, Native thiol/total thiol ratio | > | 19.6 vs 13.5 mol/L 4.0 vs 2.6 7.6 vs 4.9 53.6 vs 50.6 | CPN |
| *Piuri 2020* | [80] | CC: 30 GDM, 56 NGT | IADPSG | 26 | B-cell activating factor (BAFF) Tumor necrosis factor (TNF-a) Platelet-activating factor (PAF) Methylglyoxal (MGO) Glycated albumin (GA) | =/=/=//>/> | not significant difference not significant difference not significant difference 0.64 vs 0.25 g/mL (1.5 vs 0.95 nmol/Ml | CPN |
| *Banerjee 2020* | [81] | CC: 35 GDM, 30 NGT | IADPSG | 24-32 | Growth differentiation factor 15 (GDF-15), IL6, TNFA | > | 1061.1 VS 818.7 pg/ml 5.5 vs 2.9 pg/ mL 8.6 vs 4.9 pg/mL | CPN |
| *Tang 2019* | [82] | CC: 200 GDM, 200 NGT - 2T 130 GDM, 130 NGT - 3T | IADPSG | 26-37 | Growth differentiation factor 15 (GDF-15) | >IN 3T | 112.8 vs 78.1 ng/ml | CPN |
| *Zhao 2018* | [83] | PROSP COH: 32 NGT,29 GDM (41IGT) | NDDG | 24-28 | Hs-CRP IL-6 IL-18 TNF-a | >/>/>/+ | 5.1 vs 2.3 mg/L 5.1 vs 3.0 pg/mL 52.2 vs 22.4 pg/mL not significant difference | CPN |
| *Bahar 2020* | [84] | CC: 29 GDM, 29 NGT | IADPSG | 29-39 | IL-10, IL-35, Erythrocyte Sedimentation Rate (ESR) C-Reactive Protein (CRP) | >/>/=/=/ | Not significant difference | NSR |
| *Khosrowbegi 2018* | [85] | CC: 40 GDM, 40 NGT | IADPSG | 24-28 | TNF-α Adiponectin (ADP) Adiponectin/TNF-α ratio | >/</< | 225.1 vs 115.7 pg/mL 4.5 vs 6.37 µg/mL 4.3 vs 4.8 | CPN |
| *Mohammed, 2018* | [86] | CC: 85 GDM, 84 NGT | WHO 99 | 24-28 | TNF- α | > | 2.5 vs 2.1 pg/ml | CPN |
| *Mohammed 2018* | [87] | CC: 100 GDM, 100 NGT | WHO 99 | 24-28 | IR TNF-α | > | 3.1 vs 2.9 2.5 vs 2.0 pg/mL | CPN |
| *Zhou 2017* | [88] | CC: 180 GDM, 60 NGT | IADPSG | 24-28 | HOMA-IR TNF-α TNF Receptor 1 (TNFR1) Adiponectin (ADP) | >/>/>/< | 3.5 vs 1.4 16.6 vs 8.0 pg/mL 15.5 vs 7.1 7.3 vs 11.4 ng/L | CPN |
| *Ebru 2016* | [89] | CC: 20 NGT (27 ABN GCT) 29 GDM | C&C | 24-28 | TNF-α HsCRP Lipid profile | >/=/= | 2.7 vs 0.6 pg/mL not significant difference not significant difference | CPN |
| *Simon-muela 2015* | [90] | CC: 66 GDM, 71 NGT | NDDG | 26-30 | Tumour necrosis factor-like weak inducer of apoptosis (TWEAK), Cluster of Differentiation 163 (CD163) | </= | 237.8 vs 277.2 pg/mL not significant difference | CPN |
| *Li 2015* | [91] | CC: 35 GDM, 43 NGT | IADPSG | 24-28 | Human Cartilage Glycoprotein 39 (YKL-40) | > | 77.3 vs 50.9 ng/mL @ fasting & 63.5 vs 40.6 ng/mL @ 2 hrs | CPN |
| *Giacobbe 2018* | [92] | CC: 75 GDM, 48 NGT | IADPSG | 24-28 | High Mobility Group Box 1 (HMGB1) | > | 2.7 vs 4.5 ng/ml | CPA |
| *Ye 2017* | [93] | CC: 55 GDM, 55 NGT | IADPSG | 24-35 | Programmed cell death protein 1 (PD-1) expression on T-cell subsets | < | CD4+ T 24.0% vs 36.2% CD8+ T 26.0% vs 35.6% | CPN |
| *Pendeloski 2015* | [94] | CC: 20 GDM, 30 NGT | IADPSG | 28-36 | CD4 CD69+ CD8 CD69+ | > | 2.2 vs 1.9% 3.1 vs 2.3 % | CPN |
| *Uysal 2020* | [95] | RETR COH: 273 GDM, 455 NGT | C&C, IADPSG | 30-42 | Delta neutrophil index (DNI) | > | -2.3 vs -3.0 | CPN |
| *Ipekci 2015* | [96] | CC: 81 GDM, 38 NGT | C&C | 37-41 | Neopterin | > | 5.3 vs 3.8 nmol/l | CPN |
| **PANCREAS, LIVER AND KIDNEYS BIOMARKERS** | | | | | | | | |
| *Abbas 2018* | [97] | CC: 30 GDM, 15 NGT | C&C | 19-23/28-30 | Preptin | > | 446.3 vs 157.3 pg/mL | CPN |
| *Koroglu 2018* | [98] | CC: 25 DIET GDM, 25 INSULIN GDM 30 NGT | IADPSG | 32-38 | Pancreatic-derived factor (PANDER) | > | 448.0 vs 140.1 | CPA |
| *Adam 2018* | [99] | PROSP COH: 83 GDM, 179 NGT | IADPSG | 23- 26 | Fasting insulin Adiponectin Homeostasis Model Assessment (HOMA) Quantitative Insulin Sensitivity Check (QUICKI) | >/>/</< | 9.7 vs 6.4 uU/ml 9.3 vs 11.9 mmol/l 2.7 vs 1.3 0.6 vs 0.7 | CPN |
| *Zhu 2015* | [100] | CC: 36 GDM, 36 NGT | IADPSG | 24-28 | Insulin resistance (IR) HOMA pancreatic β-cell function (HOMA-B) Insulin sensitivity index (ISI) Insulinogenic index Corrected insulin response (CIR) | >/>/</</< | 89.2 vs 70.7 μU/mL 1.9 vs 1.3 173.8 vs 256.2 0.001 vs 0.003 15.0 vs 20.4 4.5 vs 8.7 | CPN |
| **AUTHOR, YEAR** | **REFERENCE N** | **DESIGN SAMPLE SIZE** | **GDM DIAGNOSTIC CRITERIA** | **GA** | **BIOMARKER** | **GDM vs NGT > / = / <** | **VALUES (P<0.05)** | **COMMENTS** |
| *Wang 2012* | [101] | CC: 34 IGT/GDM, 31 NGT | IADPSG | 32-36 | HOMA-IR HOMA-B AUCG ΔI30/ΔG30 ISI comp | =/>/>/</= | not significant differences 126.5 vs 112.4 24.6 vs 18.7 11.6 vs 17.8 not significant differences | CPN |
| *Saisho 2010* | [102] | RETRO COH: 57 GDM, 220 NGT | JSOG | 24-27 | Disposition index | < | * | CPN |
| *Tan 2012* | [103] | PROSP COH: 319 GDM, 2291 NGT | IADPSG | 18-32 | Gamma-glutamyl-transferase (GGT) | > | not significant differences | NSR |
| *Liu 2015* | [104] | CC: 76 GDM, 76 NGT | IADPSG | 24-26 | Butyrylcholinesterase activity γ-glutamyl transferase Cystatin C Pre-albumin | > | 6,704 vs 6,142 u/L 18.0 vs 12.9 u/L 0.057 vs 0.051 mg/L 248.1 vs 230.9 mg/L | CPN |
| *Nishimura 2018* | [105] | CC: 368 NGT, 248 GDM | IADPSG | 29-41 | Total bilirubin (TB) | < | 0.6 vs 0.66 mg/dL | CPN |
| *Zhao 2016* | [106] | CC: 111 GDM, 289 NGT | IADPSG | 24-28 | Cystatin-C. | > | 1.0 vs 0.7mg/L | CPN |
| *Yousefzadeh 2013* | [107] | CC: 30 GDM, 30 NGT | C&C | 23-37 | Cystatin-C | = | Not significant difference | NSR |
| **MUSCULOSKELETAL, CARDIOVASCULAR, ENDOTHELIAL, ADHESION MOLECULES AND PLACENTAL BIOMARKERS** | | | | | | | | |
| *Winhofer 2012* | [108] | CC: 26 GDM, 52 NGT | IADPSG | 24-28 | C-terminal cross-linking telopeptide of Type-I collagen (CTX), Osteopontin (OPN) | >/< | 0,4460.20 vs 0,2860.12 ng/ml 28,81622.12 vs.37,68619.63 ng/ml | CPN |
| *Zhang 2020* | [109] | CC: 105 GDM 46 NGT | IADPSG | 25-26 | Procollagen type 1 N-terminal propeptide (P1NP) N-terminal midfragment of osteocalcin (N-MID) β-C-terminal telopeptide of type 1 collagen (β-CTX) | =/=/> | Not significant difference Not significant difference 296.0 vs. 218.5 pg/mL | CPN FOR (β-CTX) |
| *Ural 2016* | [110] | CC: 45 GDM, 41 NGT | IADPSG | 24-28 | Irisin | < | 1.0 vs 1.3 µg/mL | CPN |
| *Zhao 2015* | [111] | CC: 61 GDM, 61 NGT | ADIPS/IADSG | 24 | Irisin | < | 213.4 vs 289.6 ng/mL | CPN |
| *Al ghazali 2020* | [112] | CC: 60 GDM, 30 NGT | IADPSG | 24-28 | Irisin | < | 71.6 VS 136. 5 ng/mL | CPN |
| *Eschler 2018* | [113] | CC: 47 GDM, 46 NGT | C&C | 26-32 | Osteoprotegerin (OPG) Prolactin Tumor necrosis factor-related apoptosis-inducing ligand (TRAIL) Hepatocyte growth factor (HGF) Plasminogen activator inhibitor type 1 (PAI-1) Tumour Necrosis Factor a (TNFA) Receptor activator of nuclear factor-kappa B ligand (RANKL) | =/=/=/=/=/=/ < | Not significant difference Not significant difference Not significant difference Not significant difference Not significant difference Not significant difference 27.95 vs 40.15 pg/mL, p=0.019). | CPN |
| *Talmor 2020* | [114] | CC: 31 GDM 35 NGT | C&C | 33-41 | Galectin-3 (Gal-3) Protein convertase subtilisin/kexin (PCSK) | >/= | * | CPN NSR |
| *Bostozun 2012* | [115] | CC: 44 GDM, 33 NGT | C&C | 16-21 | *Angiopoietin-related growth factor (AGF)* | *>* | *113.3 vs 52.3 ng/ml* | CPN |
| *Cakmak 2019* | [116] | CC: 60 GDM, 75 NGT | C&C | 24-28 | Vascular adhesion protein 1 (VAP-1) | > | 3.3 vs 1.2 ng/mL | CPA |
| *Tekin 2020* | [117] | CC: 30 NGT, 50 GDM | IADPSG | 30-32 | Signal peptide-CUB-EGF domain-containing protein (SCUBE)-1 | > | * | CPA |
| *Poniedziabek-czajkowska 2016* | [118] | CC: 25 GDM, 56 NGT | IADPSG | 26-33 | Intercellular adhesion molecule-1 (s-ICAM-1) C-reactive protein (CRP) Asymmetric dimethylarginine (ADMA) | >/>/< | 290.0 vs 232.6 ng/mL 6.5 vs 3.2 mg/L 0.4 vs 0.6 𝜇mol/L | CPN |
| *Akturk 2010* | [119] | CC: 45 GDM, 69 NGT | C&C | 32-39 | Asymmetric dimethylarginine (ADMA) hs-CRP Homocysteine (Hcy) | >/>/= | 1.4 vs 1.1 µmol/l Not significant difference 9.4 vs 6.3 mg/l | CPN |
| *Gomathi 2011* | [120] | CC 36 NGT, 26 GDM | C&C | 24-28 | Homocysteine (Hcy) Creatinine | > | 19.1 vs 8.3 µmol/L 0.9 vs 0.6 mg/dL | CPN |
| *Artunc-ulkumen 2017* | [121] | CC: 27 GDM, 30 NGT | IADPSG | 19-36 | A disintegrin and metalloproteinase with thrombospondin motifs 9 (ADAMTS-9) | < | 3.6 vs 4.6 ng/dL | CPN |
| *Demir 2019* | [122] | CC: 85 GDM, 90 NGT | IADPSG | 24-28 | Zonulin | > | 32.6 vs 12.8 ng/mL | CPN |
| *Calan 2018* | [123] | CC: 42 GDM 42 NGT | IADPSG | 25-29 | urotensin II (UII) | > | (11.6 vs. 7.6 ng/ml | CPN |
| *AUTHOR, YEAR* | **REFERENCE N** | **DESIGN SAMPLE SIZE** | **GDM DIAGNOSTIC CRITERIA** | **GA** | **BIOMARKER** | **GDM vs NGT > / = / <** | **VALUES (P<0.05)** | **COMMENTS** |
| *Li 2018* | [124] | CC: 443 GDM, 443 NGT, nested / 552 NGT and 276 GDM | IADPSG | 26–30/17-18 | trimethylamine-N-oxide (TMAO) | > | 83.0 vs 73.90 µg/L | CPN |
| *Mou 2016* | [125] | CC: 40 GDM, 40 NGT | IADPSG | 25-29 | Pigment epithelium derived factor (PEDF) | > | 5.4 vs 3.2 µg/mL | CPN |
| *Bekdemir 2015* | [126] | CC: 50 GDM, 41 NGT | C&C | 24-28 | Fibrinogen Plasminogen Von Willebrand factor (vWF) activity Coagulation Factor VIII Coagulation Factor IX | > | 503 vs 442 mg/dL 152 vs 150 227.4 vs 217.0% 171.9 vs 154.9 154.5 vs 141.1 | CPN |
| *Gorkem 2019* | [127] | PROSP COH: 76 GDM , 82 NGT | C&C | 15-28 | Placental growth factor (PlGF) | > | 0.2 vs 0.1 pg/mL | CPN |
| *Thériault 2015* | [128] | CC: 264 GDM, 528 NGT | IADPSG | 14-17 | HbA1c HsCRP SHBG | >/>/< | 5.1 vs 5.0% 6.6 vs 4.5 mg/L 357 vs 411 nmol/L | CPA |
| *Shaas 2017* | [129] | CC: 50 GMD, 50 NGT | IADPSG | 14-17 | HBA1c CRP SHBG Pregnancy-associated plasma protein A (PAPP-A) | >/>/</< | 5.9 vs 4.7 9.9 vs 7.9 mg/L 78.6 vs 88.9 mmol/l 2.5 vs 3.7 mIU/mL | CPA |

*CPA=see Table 4

* Values not available (for articles with data reported with figures with no possibility to understand the exact values)

Additional file 1b. Characteristics of included studies – exploratory phase of amniotic fluid biomarkers: Challenge Phase Available (CPA), Challenge Phase Needed (CPN) and Not Significant Results (NSR)

| **AUTHOR, YEAR** | **REFERENCE N** | **DESIGN SAMPLE SIZE** | **GDM DIAGNOSTIC CRITERIA** | **GA** | **BIOMARKER** | **GDM vs NGT > / = / <** | **VALUES (P<0.05)** | **COMMENTS** |
| --- | --- | --- | --- | --- | --- | --- | --- | --- |
| *Melekoglu 2019* | [130] | CC: 20 GDM, 20 NGT | IADPSG | 16-22 | ADAMTS 4 ADAMTS 5 IL-6 TNF-A | > | 253.5 vs 188.5 pg/mL 192.9 vs 154.8 pg/mL 136.2 vs 98.3 pg/mL 154.2 vs 86.2 pg/mL | CPN |

Additional file 1c. Characteristics of included studies – exploratory phase of ultrasound biomarkers: Challenge Phase Available (CPA), Challenge Phase Needed (CPN) and Not Significant Results (NSR)

| **AUTHOR, YEAR** | **REFERENCE N** | **DESIGN SAMPLE SIZE** | **GDM DIAGNOSTIC CRITERIA** | **GA** | **BIOMARKER** | **GDM vs NGT > / = / <** | **VALUES (P<0.05)** | **COMMENTS** |
| --- | --- | --- | --- | --- | --- | --- | --- | --- |
| **MATERNAL FEATURES** | | | | | | | | |
| *Nar, 2014* | [131] | CC: 65 GD, 64 NGT | IADPSG | 24-28 | Isovolumic relaxation time (IRT) Epicardial fat thickness (EFT) | > | 80.8 vs 71.6 ms 7.2 vs 5.6 mm | CPN |
| *D’ambrosi 2018* | [132] | CC: 56 GDM, 112 NGT | IADPSG | 24-28 | Subcutaneous adipose tissue thickness (SATT) Visceral adipose thickness (VAT) | > | 10.7 vs 9.3 mm > (values above) 10.1 vs 9.7 mm | CPN |
| *Kansu-celick 2017* | [133] | PROSP COH: 46 GDM,177 NGT | C&C | 24-28 | Subcutaneous adipose tissue thickness (SATT) | > | 19 vs 15 mm | CPA |
| *Tosun 2014* | [134] | PROSP COH: 230 NGT, 28 GDM | C&C | 20-22 | Pancreatic Body size (PS) Maternal superior mesenteric artery Doppler (SMAD), Resistance index (RI) | > | 17.5 vs 14.4 mm 4.2 vs. 3.4 > (values above) 0.7 vs 0.7 | CPN |
| *Yousefzadeh 2012* | [135] | CC: 50 GDM, 50 NGT | C&C | 20-38 | Carotid intima-media thickness (CIMT) | > | 0.65 vs 0.59 mm both times | CPN |
| **AUTHOR, YEAR** | **REFERENCE N** | **DESIGN SAMPLE SIZE** | **GDM DIAGNOSTIC CRITERIA** | **GA** | **BIOMARKER** | **GDM vs NGT > / = / <** | **VALUES (P<0.05)** | **COMMENTS** |
| *Bugatto 2018* | [136] | CC: 25 GDM, 25 NGT | NDDG | 36 | Mean Uterine Arteries (PI) | = | Not significant difference | NSR |
| *Meera 2017* | [137] | RETRO COH: 18 GDM, 72 NGT | C&C | 25-28 | Left Ventricle end-diastolic diameter (LVEDD) Left Ventricle end-systolic diameter (LVESD) Ejection fraction (EF) Posterior Wall Thickness Global longitudinal strain (GLS) Global circumferential strain (CGS) Global radial strain rate (GRSR) | =/=/=/>/>/?/? | Not significant difference Not significant difference Not significant difference 0.9 vs 0.8 cm -17.2 vs -19.8% 0.4 VS 0.4 GRSR 0.25 VS 0.19 | CPN |
| *Soydinc 2012* | [138] | CC: 42 GDM, 33 NGT | C&C | 24-36 | Left Ventricle Mass Index Dipper and non-dipper circadian variation of BP profile (nocturnal reduction in average daytime systolic BP and diastolic BP of less than 10%) | > | 102.0 vs 90.7 g/m^2^  10 dipper/32 non dipper in GDM vs 25 dipper/8 non dipper in NGT, p<0.001) | CPN |
| **FETAL FEATURES** | | | | | | | | |
| *Sovio 2016* | [139] | PROSP COH: 3898 NGT,171 GDM | WHO 99/ IADPSG | 20- 28 | Head Circumference (HC) Abdominal Circumference (AC) HC-to-AC ratio | = | Not significant difference | NSR |
| *Akiba 2020* | [140] | PROSP COH: 40 GDM, 125 NGT | JAPAN SOC OF OBST & GYNAECOLOGY | 20-37 | Fractional arm volume (AVol) fractional thigh volume (TVol) | > = | 16.4 vs 14.5 (>32 wks) Not significant difference | CPN for AVol |
| *Jin 2020* | [141] | RETRO COH: 8324 GDM, 35855 NGT | IADPSG | 22-24 | Head circumference (HC), Femur length (FL) Estimated fetal weight (EFW) | = | Not significant difference | NSR |
| *İlhan ,2018* | [142] | CC: 33 GDM, 64 NGT | IADPSG | 25-28 | Liver volume (FLV) | > | 41.5 vs 33.7 cm^3^ | CPA |
| *Showman 2019* | [143] | PROSP COH: 23 GDM, 97 NGT | NICE | 23 | Liver length (FLL) | > | 37.2 vs 33.1 | CPA (no treshold) |
| *Venkataraman 2017* | [144] | CC: 153 GDM, 178 NGT | IADPSG | 20, 32 | Anterior Abdominal Wall Thickness (AAWT) | > | 2.6 vs 2.4 mm & 4.6 vs 4.4 mm | CPN |
| *Aksoy 2015* | [145] | CC: 55 GDM, 69 NGT | IADPSG | 26-27 | Anterior Abdominal Wall Thickness (AAWT) | > | 4.1 vs 3.3 mm | CPN |
| *Tantanasis 2010* | [146] | CC: 15 NGT, 20 IGT | WHO 99 | 24-26 | Subc fat @ Head Circumference (HC) Subc fat @ Abdominal Circumference (AC) Subc fat @ Thoracic Spine (TS) | > | 5.3 vs 2.9 mm 6.6 vs 3.4 mm 7.1 vs 3.2 mm | CPA |
| *A. Yavuz 2016* | [147] | CC: 40 GDM, 40 NGT | IADPSG | 24-28 | Epicardial Fat Thickness (EFT) | > | 1.34 vs 1.31 mm | CPN |
| *Aydin 2020* | [148] | CC: 60 GDM, 60 NGT | C&C, IADPSG | 18-22 | Epicardial fat thickness (fEFT) | > | 1.0 vs 0.8 mm | CPA |
| *Aguilera 2020* | [149] | CC: 161 GDM , 483 NGT | NICE 2008 | 36-37 | Left ventricular ejection fraction Right ventricular sphericity index | < > | 0.58 vs 0.62 % 0.62 vs 0.58 | CPN |
| *Ren 2011* | [150] | CC: 169 NGT, 92 GDM (75 controlled) | C&C | 24-37 | Ventricular Wall Thickness @ End of Diastole (VWD)/ @ end of Systole (VWS) Interventricular septal thickness @ end systole (IVSS)/ @ end diastole (IVSD) Ejection Fraction (EF) Aorta/Pulmonary Artery peak systolic velocity (Ao/Pa PSV) Mitral early systolic peak velocity of annulus/late diastolic peak velocity of annulus (Mitral SA) | >than in controlled GDM | 3.0 vs 2.23 mm Left / 3.0 vs 2.3 mm Right > (values above) 3.6 vs 2.8 mm Left / 3.7 vs 2.8 mm Right 5.2 vs 4.0 mm > (values above) 4.0 vs 2.8 mm 0.31 vs 0.28 mm Left / 0.25 vs 0.24 mm Right 92.6 vs 86.7 cm/s 71.9 vs 68.2 cm/s 5.74 vs 5.17 cm/s | CPN |
| *Jatavan 2020* | [151] | CC: 43 GDM, 47 NGT | NDDG | 24-28 | Cardiac performance index (Tei index) | = | Not significant difference | NSR |
| *Dantas 2019* | [152] | CC: 115 GDM, 123 NGT | IADPSG | 26-34 | Middle cerebral artery peak systolic velocity (MCA PSV) | > | 1.0 vs 1.1 cm/s | CPN |
| **PLACENTA AND UMBILICAL CORD FEATURES** | | | | | | | | |
| *To 2009* | [153] | CC: (62 IGT) 16 GDM, 62 NGT | WHO | 38-39 | Umbilical venous (UV) Diameter UV volume flow | > | 8.2 vs 2.3 mm 8.2 vs 7.5 cm/s. | CPN |
| **AUTHOR, YEAR** | **REFERENCE N** | **DESIGN SAMPLE SIZE** | **GDM DIAGNOSTIC CRITERIA** | **GA** | **BIOMARKER** | **GDM vs NGT > / = / <** | **VALUES (P<0.05)** | **COMMENTS** |
| *Najafi 2017* | [154] | CC: 123 GDM, 123 NGT | IADPSG | 18-23, 37-41 | Umbilical coiling | < | 0.66 vs 0.74 coils/cm & 0.40 vs 0.43 coils/cm | CPN |
| *Bildaci 2017* | [155] | CC: 21 GDM, 70 NGT | IADPSG | 24-28 | Placental elasticity | = | Not significant difference | NSR |
| *Suranyi 2017* | [156] | CC: 56 GDM, 113 NGT | IADPSG | 24-37 | Placental vascularisation | < | * | CPN |
| **COMBINATION OF FEATURES** | | | | | | | | |
| *Perovic 2011* | [157] | CC: 33 GDM, 77 NGT | C&C | >/= 24 | Ultrasound gestational diabetes screening score (UGDS) Increased adipose subcutaneous tissue Increased cardiac width Increased cardiac circumference Increased placental thickness Polyhydramnion Asymmetrical macrosomy Thickened intra-ventricular septum Intensified breathing movements Immature appearance of placenta | > | % of patients 54.5 vs 5.3% 51.5 vs 5.8%, 66.7 vs 6.5% 75.8 vs 26.0% 51.5 vs 15.6%, 60.6 vs 18.2% 78.8 vs 35.1% 81.8 vs 14.3% 84.8 vs 11.7% | CPA |

Additional file 1d. Characteristics of included studies – exploratory phase of fetal/annexes’ biomarkers: Challenge Phase Available (CPA), Challenge Phase Needed (CPN) and Not Significant Results (NSR)

| **AUTHOR, YEAR** | **REFERENCE N** | **DESIGN: SAMPLE SIZE** | **GDM DIAGNOSTIC CRITERIA** | **GA** | **BIOMARKER** | **GDM vs NGT > / = / <** | **VALUES (P<0.05)** | **COMMENTS** |
| --- | --- | --- | --- | --- | --- | --- | --- | --- |
| **FETAL FEATURES** | | | | | | | | |
| *Taricco 2009* | [158] | CC: 38 GDM, 37 NGT | C&C | 38-39 | O2 saturation (SpO2) O2 content lactate glucose | </</>/> | 53.8 vs 63.2% 4.8 vs 5.5 mmol/l 1.6 vs 1.3mmol/ 3.9 vs 3.4 mmol/l | APN |
| *Teng 2017* | [159] | CC: 103 GDM, 106 NGT | IADPSG | 39-41 | ICAM-1 | = | Not significant differences | NSR |
| *Zhang 2017* | [160] | CC: 50 GDM, 50 NGT | IADSPG | 37-41 | Nesfatin-1 AFABP Leptin | >/>/> | 1.7 vs 1.2 ng/mL 20.0 vs 10.5 µg/L 9.2 vs 5.3 µg/L | APN |
| *Aslan 2012* | [161] | CC: 30 GDM, 30 NGT | NDDG | 34-41 | Apelin- 36 Nesfatin-1 | = | Not significant differences | NSR |
| *Shang 2018* | [162] | CC: 105 GDM, 105 NGT | IADPSG | 38-42 | malondialdehyde 8-isoprostane xanthine oxidase leptin resistin adiponectin | >/>/>/>/>< | * | APN |
| *Karakulak 2017* | [163] | CC: 60 GDM, 64 NGT | IADPSG | 37-41 | Ghrelin | < | 879.6 vs. 972.2 pg/ml | APN |
| *Qi 2017* | [164] | CC: 204 GDM, 204 NGT | IADPSG | 37-41 | Estradiol | < | 44.1 vs 49.9 nmol/L | APN |
| *Celik 2013* | [165] | CC: 30 GDM, 30 NGT | NDDG | 37-41 | Adropin | < | ( 1.5 vs 3.3 ng/mL, p<0.001) | APN |
| *Dube’ 2012* | [166] | CC: 18 GDM, 23 NGT | C&C | 37- 41 | C-peptide levels Glucose | > | 652 vs. 511 mmol/L 5.0 vs 4.5 mmol/L | APN |
| *Mcmanus 2014* | [167] | CC: 36 GDM,37 NGT | 75 gr OGTT fasting: <5_3 mm/l; 1 h <10_6 mm/l; 2 h <8_9 mm/l. | 28-41 | Arterial Resistin Arterial PAI-1 Venous Adiponectin Venous Resistin Venous PAI-1 | < | 57.1 vs 222.4 ng/mL 11.2 vs 21.5 64.0 vs 109.9 47.5 vs 237.0 8.4 vs 15.5 ng/mL | APN |
| **PLACENTAL FEATURES** | | | | | | | | |
| *Barke 2018* | [168] | CC: 22 GDM, 22 NGT | C&C | 35-41 | Meconium-laden macrophages CD 163 espression Total area staining positive for iron | > | * | APN |
| *Usta 2017* | [169] | CC: 23 GDM,20 NGT | C&C | 38-41 | cyclophilin A (CyPA) (score 3+ = maximum) @ trophoblast @capillary endothelium @decidua | > | 65.2 vs 10% 39.1 vs 5.0% 54.5 vs 5.9% | APN |
| *Navarro 2010* | [170] | CC: 20 GDM, 30 NGT | WHO 99 | 35-41 | Placental apolipoprotein D (apo D) 4-Hydroxynonenal (4-HNE) | > | * | APN |
| *Dairi 2020* | **[171]** | CC: 42 GDM, 42 NGT | C&C | 39-41 | Surface area of the blood vessel Placental barrier thickness/surface area of the blood vessels ratio Hofbauer cells n /surface area of the villous ratio | </</> | 107,338.0 vs 49,516.8 µ^2^ 0.003 vs 0.27 < (values above) 0.07 vs 0.0005 | APN |
| **AUTHOR, YEAR** | **REFERENCE N** | **DESIGN SAMPLE SIZE** | **GDM DIAGNOSTIC CRITERIA** | **GA** | **BIOMARKER** | **GDM vs NGT > / = / <** | **VALUES (P<0.05)** | **COMMENTS** |
| *Kadivar 2020* | [172] | CC: 117 GDM ,105 NGT | IADPSG | 38 | Umbilical cord (UC) diameter Thick edematous UC Umbilical coiling index (UCI) Persistence of central vessels | >/>/=/< | 1.41 vs. 1.28 cms 34.19% vs. 16.19% Not significant differences 32.97% vs 47.62% | APN |
| *Puransari 2020* | [173] | CC: 60 GDM, 60 NGT | C&C, IADPSG | 37-41 | Placental diameter, number of lobes, thickness, Placental weight (PW) Pllacental weight/birth weight ratio (PW/BW) Umbilical cord insertion, length, coiling, diameter | = | Not significant differences | NSR |
| *Kucuk 2009* | [174] | PROSP COH: 30 GDM (32 OAV) 242 NGT | NDDG | 37-41 | Placental weight (PW) Pllacental weight/birth weight ratio (PW/BW) | > | 694.8 vs 610.2 gr 0.21 VS 0.18 | APN |

**Additional file 2 - PRISMA checklist**

| **Section/topic** | **#** | **Checklist item** | **Reported on page #** |
| --- | --- | --- | --- |
| **TITLE** | | |  |
| Title | 1 | Identify the report as a systematic review, meta-analysis, or both. | Page 1 |
| **ABSTRACT** | | |  |
| Structured summary | 2 | Provide a structured summary including, as applicable: background; objectives; data sources; study eligibility criteria, participants, and interventions; study appraisal and synthesis methods; results; limitations; conclusions and implications of key findings; systematic review registration number. | Page 2 |
| **INTRODUCTION** | | |  |
| Rationale | 3 | Describe the rationale for the review in the context of what is already known. | Page 4 |
| Objectives | 4 | Provide an explicit statement of questions being addressed with reference to participants, interventions, comparisons, outcomes, and study design (PICOS). | Page 5,appendix2 |
| **METHODS** | | |  |
| Protocol and registration | 5 | Indicate if a review protocol exists, if and where it can be accessed (e.g., Web address), and, if available, provide registration information including registration number. | Protocol sent to Prospero, accessible upon request to authors, registration at Page 3 |
| Eligibility criteria | 6 | Specify study characteristics (e.g., PICOS, length of follow-up) and report characteristics (e.g., years considered, language, publication status) used as criteria for eligibility, giving rationale. | Page 5 |
| Information sources | 7 | Describe all information sources (e.g., databases with dates of coverage, contact with study authors to identify additional studies) in the search and date last searched. | Page 6 |
| Search | 8 | Present full electronic search strategy for at least one database, including any limits used, such that it could be repeated. | Page 6 |
| Study selection | 9 | State the process for selecting studies (i.e., screening, eligibility, included in systematic review, and, if applicable, included in the meta-analysis). | Page 6-7 |
| Data collection process | 10 | Describe method of data extraction from reports (e.g., piloted forms, independently, in duplicate) and any processes for obtaining and confirming data from investigators. | Page 7 |
| Data items | 11 | List and define all variables for which data were sought (e.g., PICOS, funding sources) and any assumptions and simplifications made. | Appendix 2, Page 19 |
| Risk of bias in individual studies | 12 | Describe methods used for assessing risk of bias of individual studies (including specification of whether this was done at the study or outcome level), and how this information is to be used in any data synthesis. | Pages 6,18 |
| Summary measures | 13 | State the principal summary measures (e.g., risk ratio, difference in means). | Pages 6,7 |
| Synthesis of results | 14 | Describe the methods of handling data and combining results of studies, if done, including measures of consistency (e.g., I^2^) for each meta-analysis. | N.A. |

| **Section/topic** | **#** | **Checklist item** | **Reported on page #** |
| --- | --- | --- | --- |
| Risk of bias across studies | 15 | Specify any assessment of risk of bias that may affect the cumulative evidence (e.g., publication bias, selective reporting within studies). | Page 7 |
| Additional analyses | 16 | Describe methods of additional analyses (e.g., sensitivity or subgroup analyses, meta-regression), if done, indicating which were pre-specified. | Page 6 |
| **RESULTS** | | |  |
| Study selection | 17 | Give numbers of studies screened, assessed for eligibility, and included in the review, with reasons for exclusions at each stage, ideally with a flow diagram. | Figure 1, pages 7-8 |
| Study characteristics | 18 | For each study, present characteristics for which data were extracted (e.g., study size, PICOS, follow-up period) and provide the citations. | Table 2 |
| Risk of bias within studies | 19 | Present data on risk of bias of each study and, if available, any outcome level assessment (see item 12). | Table 2 |
| Results of individual studies | 20 | For all outcomes considered (benefits or harms), present, for each study: (a) simple summary data for each intervention group (b) effect estimates and confidence intervals, ideally with a forest plot. | N.A. |
| Synthesis of results | 21 | Present results of each meta-analysis done, including confidence intervals and measures of consistency. | N.A. |
| Risk of bias across studies | 22 | Present results of any assessment of risk of bias across studies (see Item 15). | Page 7 |
| Additional analysis | 23 | Give results of additional analyses, if done (e.g., sensitivity or subgroup analyses, meta-regression [see Item 16]). | Page 16, Table 4 |
| **DISCUSSION** | | |  |
| Summary of evidence | 24 | Summarize the main findings including the strength of evidence for each main outcome; consider their relevance to key groups (e.g., healthcare providers, users, and policy makers). | Page 16 |
| Limitations | 25 | Discuss limitations at study and outcome level (e.g., risk of bias), and at review-level (e.g., incomplete retrieval of identified research, reporting bias). | Page 17 |
| Conclusions | 26 | Provide a general interpretation of the results in the context of other evidence, and implications for future research. | Page 17 |
| **FUNDING** | | |  |
| Funding | 27 | Describe sources of funding for the systematic review and other support (e.g., supply of data); role of funders for the systematic review. | Page 19 |

**Additional file 3. Data extraction form**

| Author Year and Title | Country of study | Aim | Pts I/E criteria * | GDM Diagnosis | Cases / controls n. | GA | Biomarker | Research Design | Methods | Main Findings | Conclusions | CASP score |
| --- | --- | --- | --- | --- | --- | --- | --- | --- | --- | --- | --- | --- |
|  |  |  |  |  |  |  |  |  |  |  |  |  |
|  |  |  |  |  |  |  |  |  |  |  |  |  |
|  |  |  |  |  |  |  |  |  |  |  |  |  |
|  |  |  |  |  |  |  |  |  |  |  |  |  |
|  |  |  |  |  |  |  |  |  |  |  |  |  |
|  |  |  |  |  |  |  |  |  |  |  |  |  |

GA= Gestational Age,

*Patients Inclusion/Exclusion criteria

**Additional file 4. Biomarkers evaluated**

| **NAME** | **SIGLE** | **CATEGORY** | **FUNCTION/SIGNIFICANCE** |
| --- | --- | --- | --- |
| *Fructosamine* | N.A. | Advanced glycation end products | Reflects albumin glycation in the last 2 weeks |
| *Glycated albumin* | GA | Advanced glycation end products | Albumin modulator of plasma oncotic pressure and transporter |
| *Glycated haemoglobin* | HbA1c | Advanced glycation end products | HbA1 modulator of erythrocyte metabolism |
| N*(epsilon)-(*carboxymethyl*)*lysine*,* | CML | Advanced glycation end products | Produced by oxidation of fructosyl-lysine |
| *Plasma glycated CD59* | pGCD59 | Advanced glycation end products | CD59 Compliment Inhibitor |
| *2-Aminobutyric Acid* | N.A. | Amino Acid | Protective Effects Against Oxidative Stress |
| *Arginine, Glycine and Methionine* | ARG, GLY, MET | Amino acid | Combine to form proteins |
| *Homocysteine* | HCYS | Amino acid | Associated with atherosclerosis |
| *Activin A* | Act-A | Cytokine | Member of the transforming growth factor beta (TGF-β) family - [Cell proliferation](https://www.sciencedirect.com/topics/medicine-and-dentistry/cell-proliferation), differentiation, wound healing, [apoptosis](https://www.sciencedirect.com/topics/medicine-and-dentistry/programmed-cell-death), and metabolism |
| *Angiopoietin-Related Growth Factor aka or angiopoietin-like protein-6* | *AGF/*ANGPTL6 | Cytokine | Stimulates metabolism and angiogenesis |
| *Apelin- 36* | N.A. | Cytokine | Related to the nutritional status and parallel insulin plasma levels |
| *B-cell activating factor* | BAFF | Cytokine | Positive regulator of B-cell function and expansion |
| *Chemerin* | N.A. | Cytokine | Adipokine: Involved in inflammation, adipogenesis, angiogenesis and energy metabolism |
| *Coiled-coil domain-containing 80* | CCDC80 | Cytokine | Increased in obesity |
| *Follistatin-like-3* | FSTL3 | Cytokine | Activin antagonist |
| *Interleukin - 6* | IL6 | Cytokine | Proinflammatory action |
| *Interluekin-10* | Il-10 | Cytokine | Anti-inflammatory |
| *Interleukin-18 (aka interferon-gamma inducing factor)* | IL-18 | Cytokine | Proinflammatory action |
| *Interleukin - 35* | IL-35 | Cytokine | Proinflammatory action |
| *Irisin* | N.A. | Cytokine | Influences metabolism |
| *Pancreatic-Derived Factor* | PANDER | Cytokine | Regulates the process of insulin release and glucose homeostasis |
| *Platelet-activating factor* | PAF | Cytokine | Stimulates platelet aggregation, vasodilation, inflammation |
| *Retinol-binding protein 4* | RBP4 | Cytokine | Adipokine: promotes insulin resistance |
| *Tumour necrosis factor A* | TNF-A | Cytokine | Proinflammatory action |
| *Tumour necrosis factor-like weak inducer of apoptosis* | TWEAK | Cytokine | Proangiogenic and proinflammatory properties |
| *Growth differentiation factor 15* | GDF-15 | Growth Factor | Regulates response to injury |
| *Hepatocyte Growth Factor* | HGF | Growth Factor | Produced by liver and placental tissues promotes cell survival and tissue regeneration in liver, pancreas, kidneys heart and placenta. |
| *Placental Growth Factor* | PlGF | Growth Factor | Placental development: angiogenesis and trophoblastic invasion of the maternal spiral arteries |
| *Adipocyte fatty acid binding protein* | AFABP | Hormone | Promotes insulin resistance |
| *Aprosin* | N.A. | Hormone | Produced in white adipose tissues, stimulates the liver to release glucose into the blood stream |
| *Atrial natriuretic peptide* | ANP | Hormone | Decrease in [systemic](https://en.wikipedia.org/wiki/Systemic_circulation) [vascular resistance](https://en.wikipedia.org/wiki/Vascular_resistance) and [central venous pressure](https://en.wikipedia.org/wiki/Central_venous_pressure) as well as an increase in [natriuresis](https://en.wikipedia.org/wiki/Natriuresis) |
| *Brain-type natriuretic peptide I* | BNP - l | Hormone | Same as ANP but 10-fold lower affinity for the receptor and twice [biological half-life](https://en.wikipedia.org/wiki/Biological_half-life) |
| *Betatrophin* | N.A. | Hormone | Controls B cells proliferation |
| *(Unconjugated) Estriol* | UE | Hormone | Free quote of Estriol, poor stimulator of uterine growth and plasminogen activator activity |
| *Ghrelin* | N.A. | Hormone | Regulation of eating behaviour and body weight |
| *Insulin* | N.A. | Hormone | Facilitates cellular glucose uptake, regulates metabolism/promotes cell division and growth |
| *Leptin* | N.A. | Hormone | Produced by adipose cells and enterocytes inhibits hunger |
| *Prolactin* | PRL | Hormone | Promotes and sustains lactation and glucose homeostasis |
| *Preptin* | N.A. | Hormone | Co-secreted with insulin and amylin from the pancreatic beta-cells, increases glucose-mediated insulin secretion |
| *Resistin (AKA adipose tissue-specific secretory factor or C/EBP-epsilon-regulated myeloid-specific secreted cysteine-rich protein)* | ADSF/ XCP1 | Hormone | [Adipose derived](https://en.wikipedia.org/wiki/Adipose-derived_hormone) involved in obesity and T2DM development |
| **NAME** | **SIGLE** | **CATEGORY** | **FUNCTION/SIGNIFICANCE** |
| *Thyroid Hormones: Thyroid Stimulating Hormone Triiodothyronine Thyroxine* | TSH FT3 FT4 | Hormone | . Stimulates the thyroid to produce T4/T3 Active form– activate the metabolism Transformed into T3 |
| *Lysophospholipids (Lysophosphatidylethanolamine-LPE; Lysophosphatidylcholine-LPC; Lysophosphatidylinositol – LPI; Lysophosphoserune- LPS; Lysophosphatidic acid –LPA)* | LPLs | Lipid molecules | Extra/intra-cellular mediators |
| *Triglycerides* | N.A. | Lipid molecules | Fat storage |
| *Asymmetric Dimethylarginine* | ADMA | Others | *Enzyme* Inhibitor: Inhibit NO synthesis impairing endothelial function and promoting atherosclerosis |
| *Bile Acids (dihydroxy conjugated, trihydroxy unconjugated and sulfated bile acids)* | N.A. | Others | Lipid derived product : Facilitate [digestion of dietary fats and oils](https://en.wikipedia.org/wiki/Digestion#Fat_digestion) |
| *3-carboxy-4-methyl-5-propyl-2-furanpropanoic acid* | CMPF | Others | Organic compound : Uremic toxin |
| *Disulfide* | N.A. | Others | Protein component: Disulfide bonds play a key role in stabilizing protein structures, with disruption strongly associated with loss of protein function and activity due to oxidation |
| *Endogenous NOS Inhibitor* | N.A. | Others | *Enzyme* Inhibitor : Contributes to endothelial dysfunction |
| *Iron* | N.A. | Others | Element: Haemoglobin and Myoglobin production |
| *Itaconic acid* | IA | Others | Organic compound : Possess antibacterial activities |
| *Long-chain polyunsaturated fatty acids (including omega-3 (docosahexaenoic acid or DHA)* | (LCPUFAs) | Others | Organic compound : Required for normal growth, to support immunity, and can improve cardiovascular and brain health. |
| *Neopterin* | N.A. | Others | Nucleotide derived: Marker for the activation of the immune system and neuroinflammation |
| *T Lymphocytes* | *CD4, CD69, And CD8 T* | Others | Cells: Components of the adaptive immune system |
| *Total Bilirubin* | TB | Others | Lipid derived Pigment : Shown to possess important functions as an antioxidant, it allows the excretion of heme, from haemoglobin, myoglobin, and various P450 enzymes. |
| *8 Isoprostane (of which 8-iso-prostaglandin (8-iso-PGF2𝛼),)* | 8Isop | Oxidation/ Peroxidation product | Marker of oxidative stress |
| *Malondialdehyde* | MDA | Oxidation/ Peroxidation Product | Marker of oxidative stress |
| *Methylglyoxal* | MGO | Oxidation/ Peroxidation Product | Oxidizing substance leades to oxidative stress, cellular aging, DNA mutations, and apoptosis |
| *Nitrotyrosine* | NT | Oxidation/ Peroxidation product | Marker of cell damage, inflammation as well as NO (nitric oxide) production. |
| *Oxidised low-density lipoprotein* | Ox-LDL | Oxidation/ Peroxidation Product | Atherosclerosis |
| *Protein Carbonyl* | PCO | Oxidation/ Peroxidation product | Markers of oxidative stress |
| *Protein Hydroperoxides* | P-OOH | Oxidation/ Peroxidation Product | Direct reactivity with a variety of biomolecules and the ability to decompose to free radicals |
| *Trimethylamine-N-Oxide* | TMAO | Oxidation/ Peroxidation product | Independent risk factor for the development of atherosclerosis and cardiovascular diseases |
| *Adropin* | N.A. | Peptide | Maintenance of energy homeostasis and insulin secretion |
| *β-C-terminal telopeptide of type 1 collagen* | β-CTX | Peptide | Marker of bone resorption |
| *Copeptin* | CT-proAVP | Peptide | Vasopressin surrogate marker |
| *C-peptide* | N.A. | Peptide | reflects the insulin-secretory activity of pancreatic *β*-cells |
| *C-Terminal Cross-Linking Telopeptide Of Type-I Collagen* | CTX | Peptide | Marker of bone resorption |
| *Galanin* | N.A. | Peptide | Neuronal inhibitor |
| *Nesfatin-1* | N.A. | Peptide | Regulation of hunger and fat storage |
| *N-terminal midfragment of osteocalcin* | N-MID | Peptide | Marker of bone formation |
| *Procollagen type 1 N-terminal propeptide* | P1NP | Peptide | Marker of bone formation |
| *Spexin* | N.A. | Peptide | Involvement in energy homeostasis and food intake |
| *Urotensin II* | UII | Peptide | Modulation of vessels dynamics and insulin resistance |
| *Adiponectin* | N.A. | Protein | Adipocyte-specific involved in insulin resistance and atherosclerosis |
| **NAME** | **SIGLE** | **CATEGORY** | **FUNCTION/SIGNIFICANCE** |
| *A Disintegrin And Metalloproteinase With Thrombospondin Motifs 9* | ADAMTS-9 | Protein | Extracellular matrix re-modelling, angiogenesis, fibrosis, and coagulation |
| *Apolipoprotein A1* | ApoA1 | Protein | Primary protein component of high-density lipoprotein (HDL) |
| *Apolipoprotein D* | apo D | Protein | Antioxidant |
| *Chitinase-3 like-protein-1* | YKL-40 | Protein | Marker of inflammation - binds to chitin, heparin, and hyaluronic acid |
| *Cluster of Differentiation 163* | Cd163 | Protein | Haemoglobin scavenger receptor is a macrophage specific protein characteristic of tissues responding to inflammation |
| *C reactive Protein* | CRP | Protein | Marker of inflammation |
| *Cyclophilin A* | CyPA | Protein | Mediates intracellular protein folding, intracellular and extracellular trafficking, and protein-protein interaction; immunosuppressive effect. |
| *Cystatin C* | Cys-C | Protein | Marker of glomerular filtration |
| *Ficolin 3* | FCN 3 | Protein | Activates the complement pathway |
| *Ferritin* | N.A. | Protein | Iron storage |
| *Fibrinogen aka Factor I* | N.A. | Protein | Involved coagulation, revascularization and [wound healing](https://en.wikipedia.org/wiki/Wound_healing) |
| *Follistatin* | FS | Protein | Binding and bio neutralization of members of the TGF-β superfamily |
| *Galectin-3* | Gal-3 | Protein | Mediator of cell damage: pro-fibrotic and pro-inflammatory properties |
| *Glutathione Peroxidase-3* | GPX-3 | Protein | Antioxidant |
| *High Mobility Group Box 1* | HMGB1 | Protein | Nuclear protein that organizes the DNA and regulates transcription |
| *Human Cartilage Glycoprotein 39* | YKL-40 | Protein | Plays important roles in inflammation, extracellular remodelling, fibrosis, and angiogenesis |
| *Neuregulin 4* | NRG4 | Protein | Signalling |
| *Osteopontin* | OPN | Protein | Involved in physiological and pathological bone mineralization and inflammatory disorders |
| *Osteoprotegerin aka Osteoclastogenesis inhibitory factor or Tumour necrosis factor receptor superfamily member 11B* | OPG/OCIF/TNFRSF11B | Protein | Regulates bone density and inhibits apoptosis of specific cells |
| *Paraoxonase-1* | PON1 | Protein | Anti-inflammatory & Antioxidant |
| *Pigment Epithelium Derived Factor* | PEDF | Protein | Related to insulin sensitivity and involved in the occurrence and complications of diabetes mellitus (nephropathy and retinopathy) |
| *Plasminogen* | N.A. | Protein | Plasmin precursor |
| *Plasminogen Activator Inhibitor Type 1* | PAI-1 | *Protein* | Involved in tumorigenesis, angiogenesis, wound healing, ovulation, and regulation of anti-fibrinolytic activity of the plasma. |
| *Pre-Albumin (transthyretin)* |  | Protein | Carries thyroxine (the main thyroid hormone) and vitamin A |
| *Protein convertase subtilisin/kexin* | PCSK | Protein | Induce degradation of LDL receptors in the lysosome of hepatocytes reducing ldl metabolism and generating inflammation |
| *Programmed Cell Death Protein 1* | PD-1/ CD279 | Protein | Down-regulates the immune system and promotes self-tolerance by suppressing T cell |
| *Receptor Activator Of Nuclear Factor-Kappa B Ligand aka Osteoprotegerin ligand* | RANKL/ OPGL | Protein | Stimulates osteoclast differentiation and activity, as well as prevention of osteoclast apoptosis |
| *Secreted frizzled-related protein 4* | SFRP4 | Protein | Regulates bone morphogenesis/ adult uterine morphology and function/ apoptosis during ovulation |
| *Sex hormone binding globulin* | SHBG | Protein | Hormones transport |
| *Signal peptide-CUB-EGF domain-containing protein* | SCUBE-1 | Protein | Expression of hypoxia, endothelial dysfunction, and vascular injury |
| *Soluble Adhesion Molecules* | sICAM-1, sVCAM-1 | Protein | Influence the binding of monocytic cells to vascular endothelium in inflammatory processes |
| *TNF-a soluble receptors* | sTNFaRs: sTNF-R1 and sTNF-R2 | Protein | Their concentration is proportional to previous TNFa action and remain elevated in plasma for longer periods |
| *Transferrin* | TfR | Protein | Iron metabolism |
| *Transferrin receptor* | sTfR | Protein | Cellular iron acquisition |
| *Transthyretin* | TTR | Protein | Transport protein in serum/cerebrospinal fluid for thyroxine (T_4_) and retinol-binding protein bound to retinol |
| *Tumor necrosis factor-related apoptosis-inducing ligand* | TRAIL | Protein | Expressed on the surface of natural killer and T cells, macrophages, and dendritic cells. |
| *Vascular adhesion protein 1* | VAP-1 | *Protein* | Surface adhesion molecule, mediates leukocyte extravasation and contributes to oxidative stress |
| *Visceral adipose tissue derived serine [protease](https://www.sciencedirect.com/topics/pharmacology-toxicology-and-pharmaceutical-science/proteinase) inhibitor (aka Serpin A12)* | VASPIN | Protein | Improves glucose tolerance and reduces food intake |
| *Von Willerbrand Factor* | VWF | Protein | Coagulation |
| *Zonulin* | N.A. | Protein | Biomarker of impaired gut barrier function for several autoimmune, neurodegenerative, and tumoral diseases |
| *Adiponectin/TNF-α ratio* | N.A. | Score/ratio/index | Expression of anti vs pro inflammatory molecules |
| *Body Mass Index* | BMI | Score/ratio/index | =weight/height^2^ |
| *Delta Neutrophil Index* | DNI | Score/ratio/index | Corresponds to the fraction of circulating immature granulocytes, linked to infection |
| **NAME** | **SIGLE** | **CATEGORY** | **FUNCTION/SIGNIFICANCE** |
| *Disposition Index* | DI | Score/ratio/index | Product of insulin sensitivity times the amount of insulin secreted in response to blood glucose levels. |
| *Fasting Glucose (Plasma/Blood)* | FG – FPG/FBG | Score/ratio/index | Level of blood glucose at fasting |
| *HOMA pancreatic β-cell function* | HOMA-B | Score/ratio/index | Estimates steady state beta cell function (%B) as percentages of a normal reference population |
| *Homeostatic model assessment for Insulin resistance - index* | HOMA-IR | Score/ratio/index | Resistance index derived by fasting glucose and insulin |
| *Insulin sensitivity index* | ISI | Score/ratio/index | Function of the measured glucose and insulin levels |
| *Insulinogenic index* | IGI | Score/ratio/index | Index of insulin secretion derived from OGTT = δ insulin (0-30 min)/δ glucose (0-30 min) |
| *Neutrophil-to-lymphocyte ratio and* | NLR | Score/ratio/index | Marker of inflammation |
| *Platelet-to-lymphocyte ratio* | PLR | Score/ratio/index | Marker of inflammation |
| *Quantitative Insulin Sensitivity Check* | QUICKI | Score/ratio/index | = 1/[log(I_0_) + log(G_0_)] where I_0_ is the fasting insulin, and G_0_ is the fasting glucose |
| *Serum Transferrin/ferretin ratio* | sTfR-F ratio | Score/ratio/index | Covers the full spectrum of iron homeostasis, from normal, healthy iron stores to mild or substantial functional iron deficiency |
| *Total Antioxidant Capacity* | TAC | Score/ratio/index | Measure of the number of free radicals scavenged by a test solution |
| *Transferrin saturation* | TS | Score/ratio/index | Serum iron/ total iron-binding capacity of available transferrin (TIBC) |
| *Triglyceride to high-density lipoprotein cholesterol ratio* | TG/HDL-C | Score/ratio/index | High levels associated with obesity, metabolic syndrome, and insulin resistance |
| *Triglyceride-glucose index* | TyG | Score/ratio/index | Insulin resistance Index |
| *Butyrylcholinesterase Activity* | BChE activity | Score/ratio/index – Miscellaneous markers | Used as a liver function test |
| *Disuplhide/Native Thiol,* | N.A. | Score/ratio/index – Miscellaneous markers | Expression of oxidation |
| *Disulfide/Total Thiol* | N.A. | Score/ratio/index – Miscellaneous markers | Expression of oxidation |
| *High-sensitivity C-reactive protein* | Hs-CRP | Score/ratio/index – Miscellaneous markers | Finds lower levels of CRP - Cardiovascular disease |
| *Isovolumic Relaxation Time* | IVRT | Score/ratio/index – Miscellaneous markers | Time interval between the end of aortic ejection and the beginning of ventricular filling in ms. |
| *Mean Platelet Volume* | MPV | Score/ratio/index – Miscellaneous markers | Average platelet size |
| *Native thiol/total thiol ratio* | N.A. | Score/ratio/index – Miscellaneous markers | Expression of oxidation |
| *Oxidative Stress Index* | OSI | Score/ratio/index – Miscellaneous markers | Ratio of TOS to TAS (Total antioxidant status) |
| *Placental barrier thickness/surface area of the blood vessels ratio* | N.A. | Score/ratio/index – Miscellaneous markers | Adaptation to diabetes-related hypoxia |
| *Placental Hoffbauer cells n /surface area of the villous ratio* | N.A. | Score/ratio/index – Miscellaneous markers | Adaptation to diabetes-related hypoxia |
| *Placental Surface area of the blood vessel,* | N.A. | Score/ratio/index – Miscellaneous markers | Adaptation to diabetes-related hypoxia |
| *Placental weight/birth weight ratio (PW/BW)* | N.A. | Score/ratio/index – Miscellaneous markers | Reflex of the growth-promoting environment of GDM |
| *Tei index* | N.A. | Score/ratio/index – Miscellaneous markers | Marker of cardiac function: sum of the isovolumic contraction and relaxation times divided by the ejection time |
| *Total Oxidative Stress* | TOS | Score/ratio/index – Miscellaneous markers | Marker of the overall oxidation state of the body |
| *Umbilical coiling index* | UCI | Score/ratio/index – Miscellaneous markers | Total number of coils divided by the total length of the cord |
| **NAME** | **SIGLE** | **CATEGORY** | **FUNCTION/SIGNIFICANCE** |
| *Estimated fetal weight* | EFW | Ultrasound measure | Derived with Hadlock formula: HC, AC, and FL |
| *Femur length* | FL | Ultrasound measure | Basic biometric parameter used to assess fetal size |
| *Epicardial Fat Thickness* | EFT | Ultrasound measure | Echo-free space between the outer wall of the myocardium and the visceral layer of the pericardium |
| *Fetal Liver length* | FLL | Ultrasound measure | Measure of the fetal liver |
| *Fractional arm volume* | AVol | Ultrasound measure | Cylindrical limb volume based on 50% of the fetal  humeral diaphysis length |
| *Fractional thigh volume* | TVol | Ultrasound measure | Cylindrical limb volume based on 50% of the fetal  femoral diaphysis length |
| *Head circumference* | HC | Ultrasound measure | Basic biometric parameter used to assess fetal size |
| *Cobalamin* | Vit B12 | Vitamin | Involved in cells metabolism |
| *Vitamin D* | 25(OH)D | Vitamin | Calcium metabolism and anti-inflammatory action |

N.A.=Not available

References

1. Ryser Ruetschi, J., et al., *Fasting glycaemia to simplify screening for gestational diabetes.* BJOG, 2016. **123**(13): p. 2219-2222.

2. Trujillo, J., et al., *Fasting plasma glucose to avoid a full OGTT in the diagnosis of gestational diabetes.* Diabetes Res Clin Pract, 2014. **105**(3): p. 322-6.

3. d'Emden, M., et al., *Development of a fasting blood glucose-based strategy to diagnose women with gestational diabetes mellitus at increased risk of adverse outcomes in a COVID-19 environment.* PLoS One, 2020. **15**(12): p. e0243192.

4. Anjalakshi, C., et al., *A single test procedure to diagnose gestational diabetes mellitus.* Acta Diabetol, 2009. **46**(1): p. 51-4.

5. Ghosh, P., et al., *Plasma Glycated CD59, a Novel Biomarker for Detection of Pregnancy-Induced Glucose Intolerance.* Diabetes Care, 2017. **40**(7): p. 981-984.

6. Rajput, R., et al., *Utility of HbA1c for diagnosis of gestational diabetes mellitus.* Diabetes Res Clin Pract, 2012. **98**(1): p. 104-7.

7. Kwon, S.S., et al., *HbA1c for diagnosis and prognosis of gestational diabetes mellitus.* Diabetes Res Clin Pract, 2015. **110**(1): p. 38-43.

8. Renz, P.B., et al., *HbA1c Test as a Tool in the Diagnosis of Gestational Diabetes Mellitus.* PLoS One, 2015. **10**(8): p. e0135989.

9. Siricharoenthai, P. and V. Phupong, *Diagnostic accuracy of HbA1c in detecting gestational diabetes mellitus.* J Matern Fetal Neonatal Med, 2020. **33**(20): p. 3497-3500.

10. Khan, S.H., et al., *Role of HbA1c in diagnosis of gestational diabetes mellitus.* J Pak Med Assoc, 2020. **70**(10): p. 1731-1736.

11. Lai, Y., et al., *The diagnostic accuracy of HbA1c in detecting gestational diabetes mellitus among Chinese pregnant individuals.* Ann Transl Med, 2020. **8**(16): p. 1014.

12. Rayis, D.A., et al., *Reliability of glycosylated hemoglobin in the diagnosis of gestational diabetes mellitus.* J Clin Lab Anal, 2020. **34**(10): p. e23435.

13. Huang, Y., et al., *Glycated albumin is an optimal biomarker for gestational diabetes mellitus.* Exp Ther Med, 2015. **10**(6): p. 2145-2149.

14. Zhu, J., et al., *The diagnostic value of glycated albumin in gestational diabetes mellitus.* J Endocrinol Invest, 2018. **41**(1): p. 121-128.

15. Li, S. and H. Yang, *Relationship between advanced glycation end products and gestational diabetes mellitus.* J Matern Fetal Neonatal Med, 2019. **32**(17): p. 2783-2789.

16. Bartakova, V., et al., *Serum carboxymethyl-lysine, a dominant advanced glycation end product, is increased in women with gestational diabetes mellitus.* Biomed Pap Med Fac Univ Palacky Olomouc Czech Repub, 2016. **160**(1): p. 70-5.

17. Gingras, V., et al., *Mid-Pregnancy Fructosamine Measurement-Predictive Value for Gestational Diabetes and Association with Postpartum Glycemic Indices.* Nutrients, 2018. **10**(12).

18. A., Ç., *Mean Platelet Volume in Women with Gestational Diabetes.* Turkish Journal of Endocrinology and Metabolism, 2016. **20**: p. 48-53.

19. Sargin, M.A., et al., *Neutrophil-to-lymphocyte and platelet-to-lymphocyte ratios: are they useful for predicting gestational diabetes mellitus during pregnancy?* Ther Clin Risk Manag, 2016. **12**: p. 657-65.

20. Yilmaz, H., et al., *Benefits of the neutrophil-to-lymphocyte ratio for the prediction of gestational diabetes mellitus in pregnant women.* Exp Clin Endocrinol Diabetes, 2014. **122**(1): p. 39-43.

21. Aktulay, A., et al., *Gestational Diabetes Mellitus Seems to Be Associated with Inflammation.* Acta Clin Croat, 2015. **54**(4): p. 475-8.

22. Yuan, X.S., et al., *Increased secreted frizzled-related protein 4 and ficolin-3 levels in gestational diabetes mellitus women.* Endocr J, 2018. **65**(4): p. 499-508.

23. Dudzik, D., et al., *Metabolic fingerprint of Gestational Diabetes Mellitus.* J Proteomics, 2014. **103**: p. 57-71.

24. Chen, X., et al., *Metabolomic biomarkers and novel dietary factors associated with gestational diabetes in China.* Metabolomics, 2018. **14**(11): p. 149.

25. Zheng, S., et al., *Metabolic Profiling of Plasma in Gestational Diabetes Mellitus Using Liquid Chromatography and Q-TOF Mass Spectrometry.* Clin Lab, 2017. **63**(7): p. 1045-1055.

26. De Seymour, J.V., et al., *Early pregnancy metabolite profiling discovers a potential biomarker for the subsequent development of gestational diabetes mellitus.* Acta Diabetol, 2014. **51**(5): p. 887-90.

27. Zhao, J.P., et al., *Longitudinal circulating concentrations of long-chain polyunsaturated fatty acids in the third trimester of pregnancy in gestational diabetes.* Diabet Med, 2016. **33**(7): p. 939-46.

28. Yi, J., et al., *Increased serum 3-carboxy-4-methyl-5-propyl-2-furanpropanoic acid (CMPF) levels are associated with glucose metabolism in Chinese pregnant women.* J Endocrinol Invest, 2018. **41**(6): p. 663-670.

29. Wang, D., et al., *The associations between triglyceride to high-density lipoprotein cholesterol ratios and the risks of gestational diabetes mellitus and large-for-gestational-age infant.* Clin Endocrinol (Oxf), 2015. **83**(4): p. 490-7.

30. Sanchez-Garcia, A., et al., *Diagnostic accuracy of the triglyceride-glucose index for gestational diabetes screening: a practical approach.* Gynecol Endocrinol, 2020. **36**(12): p. 1112-1115.

31. Alyas, S., et al., *Early pregnancy biochemical markers of placentation for screening of gestational diabetes mellitus (GDM).* Diabetes Metab Syndr, 2019. **13**(4): p. 2353-2356.

32. Retnakaran, R., et al., *Serum apoA1 (Apolipoprotein A-1), Insulin Resistance, and the Risk of Gestational Diabetes Mellitus in Human Pregnancy-Brief Report.* Arterioscler Thromb Vasc Biol, 2019. **39**(10): p. 2192-2197.

33. Ghaneei, A., et al., *Increased serum oxidized low-density lipoprotein levels in pregnancies complicated by gestational diabetes mellitus.* Iran J Reprod Med, 2015. **13**(7): p. 421-4.

34. Yuan, X.S., et al., *Ficolin-3/adiponectin ratio for the prediction of gestational diabetes mellitus in pregnant women.* J Diabetes Investig, 2018. **9**(2): p. 403-410.

35. Bagci, *Associations between serum levels of adiponectin and*

*resistin and metabolic parameters in pregnant women*

*with gestational diabetes mellitus.* Clinical and Experimental

Obstetrics & Gynecology, 2018. **45**: p. 539-543.

36. Karasek, D., et al., *Circulating levels of selected adipokines in women with gestational diabetes and type 2 diabetes.* Journal of Applied Biomedicine, 2020. **18**(2): p. 54-60.

37. Sengul, O.B., et al., *Investigation of the correlation between 100 gram oral glucose tolerance test results and maternal leptin levels during pregnancy.* J Turk Ger Gynecol Assoc, 2009. **10**(3): p. 158-61.

38. Fatima, S.S., et al., *Elevated levels of chemerin, leptin, and interleukin-18 in gestational diabetes mellitus.* J Matern Fetal Neonatal Med, 2017. **30**(9): p. 1023-1028.

39. Ortega-Senovilla, H., et al., *Gestational diabetes mellitus causes changes in the concentrations of adipocyte fatty acid-binding protein and other adipocytokines in cord blood.* Diabetes Care, 2011. **34**(9): p. 2061-6.

40. Kralisch, S., et al., *Serum levels of adipocyte fatty acid binding protein are increased in gestational diabetes mellitus.* Eur J Endocrinol, 2009. **160**(1): p. 33-8.

41. Wang, X., et al., *Expression and correlation of Chemerin and FABP4 in peripheral blood of gestational diabetes mellitus patients.* Exp Ther Med, 2020. **19**(1): p. 710-716.

42. Liu, L., et al., *A novel association of CCDC80 with gestational diabetes mellitus in pregnant women: a propensity score analysis from a case-control study.* BMC Pregnancy Childbirth, 2020. **20**(1): p. 53.

43. Ning, H., et al., *Plasma fatty acid-binding protein 4 (FABP4) as a novel biomarker to predict gestational diabetes mellitus.* Acta Diabetol, 2016. **53**(6): p. 891-898.

44. Zhang, Y., et al., *Changes in serum adipocyte fatty acid-binding protein in women with gestational diabetes mellitus and normal pregnant women during mid- and late pregnancy.* J Diabetes Investig, 2016. **7**(5): p. 797-804.

45. Su, Y.X., et al., *Increased serum retinol-binding protein-4 levels in pregnant women with and without gestational diabetes mellitus.* Diabetes Metab, 2010. **36**(6 Pt 1): p. 470-5.

46. Tawfeek, M.A., et al., *Sex hormone binding globulin as a valuable biochemical marker in predicting gestational diabetes mellitus.* BMC Womens Health, 2017. **17**(1): p. 18.

47. Pan, R., et al., *Betatrophin for diagnosis and prognosis of mothers with gestational diabetes mellitus.* J Int Med Res, 2019. **47**(2): p. 710-717.

48. Ebert, T., et al., *Betatrophin levels are increased in women with gestational diabetes mellitus compared to healthy pregnant controls.* Eur J Endocrinol, 2015. **173**(1): p. 1-7.

49. Erol, O., et al., *Evaluation of circulating betatrophin levels in gestational diabetes mellitus.* Gynecol Endocrinol, 2015. **31**(8): p. 652-6.

50. Zhong, L., et al., *Continuous elevation of plasma asprosin in pregnant women complicated with gestational diabetes mellitus: A nested case-control study.* Placenta, 2020. **93**: p. 17-22.

51. Gorar, S., et al., *Comparison of thyroid function tests and blood count in pregnant women with versus without gestational diabetes mellitus.* J Obstet Gynaecol Res, 2017. **43**(5): p. 848-854.

52. AMIRIAN, A., et al., *Predictive Power of Unconjugated Estriol in Diagnosis of Gestational Diabetes: A Cohort Study.* IRANIAN RED CRESCENT MEDICAL JOURNAL (IRCMJ), 2019. **21**(11): p. -.

53. Liu, M., Y. Chen, and D. Chen, *Association between transthyretin concentrations and gestational diabetes mellitus in Chinese women.* Arch Gynecol Obstet, 2020. **302**(2): p. 329-335.

54. Ebert, T., et al., *Serum Levels of Copeptin are Decreased in Gestational Diabetes Mellitus.* Exp Clin Endocrinol Diabetes, 2016. **124**(4): p. 257-60.

55. Zhang, Z., et al., *Association between circulating levels of galanin and pre-pregnancy body mass index in patients with gestational diabetes mellitus.* Eat Behav, 2015. **19**: p. 57-60.

56. Zhang, Z., et al., *Endogenous galanin as a novel biomarker to predict gestational diabetes mellitus.* Peptides, 2014. **54**: p. 186-9.

57. Nergiz, S., et al., *Circulating galanin and IL-6 concentrations in gestational diabetes mellitus.* Gynecol Endocrinol, 2014. **30**(3): p. 236-40.

58. Naf, S., et al., *Serum activin A and follistatin levels in gestational diabetes and the association of the Activin A-Follistatin system with anthropometric parameters in offspring.* PLoS One, 2014. **9**(4): p. e92175.

59. Yuksel, M.A., et al., *Maternal serum atrial natriuretic peptide (ANP) and brain-type natriuretic peptide (BNP) levels in gestational diabetes mellitus.* J Matern Fetal Neonatal Med, 2016. **29**(15): p. 2527-30.

60. Kucukler, F.K., et al., *Low level of Nesfatin-1 is associated with gestational diabetes mellitus.* Gynecol Endocrinol, 2016. **32**(9): p. 759-761.

61. Ademoglu, E.N., et al., *Serum nesfatin-1 levels are decreased in pregnant women newly diagnosed with gestational diabetes.* Arch Endocrinol Metab, 2017. **61**(5): p. 455-459.

62. Mierzynski, R., et al., *Nesfatin-1 and Vaspin as Potential Novel Biomarkers for the Prediction and Early Diagnosis of Gestational Diabetes Mellitus.* Int J Mol Sci, 2019. **20**(1).

63. Yavuzkir, S., et al., *Maternal and umbilical cord blood subfatin and spexin levels in patients with gestational diabetes mellitus.* Peptides, 2020. **126**: p. 170277.

64. Kurek Eken, M., et al., *Clinical significance of neuregulin 4 (NRG4) in gestational diabetes mellitus.* Gynecol Endocrinol, 2018. **34**(7): p. 605-608.

65. Butt, *Low Serum Cobalamin is a Risk Factor for*

*Gestational Diabetes.* Pakistan J. Zool.,, 2017. **49**: p. 1963-1968,.

66. Khosrowbeygi, A. and H. Ahmadvand, *Maternal circulating status of vitamin D, adiponectin and lipid ratios in gestational diabetes mellitus.* Clinical Diabetology, 2020. **9**(5): p. 321-327.

67. Yaqiong, L., et al., *Study on the levels of 25(OH)D, inflammation markers and glucose and fat metabolism indexes in pregnant women of Han nationality in Jiangsu province with gestational diabetes mellitus.* Medicine (Baltimore), 2020. **99**(35): p. e21654.

68. Sarker, M.R., et al., *Role of Maternal Iron Status in the Pathogenesis Of Gestational Diabetes Mellitus.* 2014. **40**: p. 55-60.

69. Afkhami-Ardekani, M. and M. Rashidi, *Iron status in women with and without gestational diabetes mellitus.* J Diabetes Complications, 2009. **23**(3): p. 194-8.

70. Alam, F.S., H., Khuwaja, S. et al. , *Implication of soluble transferrin receptor and ferritin ratio in gestational diabetes.* Int J Diabetes Dev Ctries, 2018. **38**: p. 42-46.

71. Rueangdetnarong, H., et al., *Comparisons of the oxidative stress biomarkers levels in gestational diabetes mellitus (GDM) and non-GDM among Thai population: cohort study.* Endocr Connect, 2018. **7**(5): p. 681-687.

72. Li, H., et al., *Plasma Markers of Oxidative Stress in Patients with Gestational Diabetes Mellitus in the Second and Third Trimester.* Obstet Gynecol Int, 2016. **2016**: p. 3865454.

73. Gelisgen, R., et al., *Protein oxidation markers in women with and without gestational diabetes mellitus: a possible relation with paraoxonase activity.* Diabetes Res Clin Pract, 2011. **94**(3): p. 404-9.

74. Lewandowski, K.C., et al., *Raised concentrations of lipid peroxidation products (LPO) in pregnant women with impaired glucose tolerance.* Ann Agric Environ Med, 2014. **21**(2): p. 429-34.

75. Beyazit, F., K.Ö. Karacaer, and H. Turkon, *The association of oxidative stress with serum irisin and betatrophin in pregnant women with gestational diabetes mellitus.* Clinical Diabetology, 2020. **9**(5): p. 328-334.

76. Ma, S.G., et al., *Evaluation of serum ischemia-modified albumin levels in pregnant women with and without gestational diabetes mellitus.* Gynecol Endocrinol, 2012. **28**(11): p. 837-40.

77. Ozler, S., et al., *Are serum levels of ADAMTS5, TAS and TOS at 24-28 gestational weeks associated with adverse perinatal outcomes in gestational diabetic women?* J Obstet Gynaecol, 2020. **40**(5): p. 619-625.

78. Parast, V.M. and Z. Paknahad, *Antioxidant Status and Risk of Gestational Diabetes Mellitus: a Case-Control Study.* Clin Nutr Res, 2017. **6**(2): p. 81-88.

79. Cakina, S., B. Aydin, and F. Beyazit, *Evaluation of thiol/disulfide homeostasis in patients with gestational diabetes mellitus.* Gynecol Endocrinol, 2020. **36**(11): p. 1006-1009.

80. Piuri, G., et al., *Methylglyoxal, Glycated Albumin, PAF, and TNF-alpha: Possible Inflammatory and Metabolic Biomarkers for Management of Gestational Diabetes.* Nutrients, 2020. **12**(2).

81. Banerjee, S., et al., *A study of serum growth differentiation factor 15 in Indian women with and without gestational diabetes mellitus in the third trimester of pregnancy and its association with pro-inflammatory markers and glucose metabolism.* Diabetology International, 2020: p. 1-6.

82. Tang, M., et al., *Serum growth differentiation factor 15 is associated with glucose metabolism in the third trimester in Chinese pregnant women.* Diabetes Res Clin Pract, 2019. **156**: p. 107823.

83. Zhao, X., et al. *Correlation between inflammatory markers ( hs-CRP , TNF0± , IL-1#² , IL-6 , IL-18 ) , glucose intolerance , and gestational diabetes mellitus in pregnant women*. 2018.

84. A, B., *Anti-Inflammatory Markers IL-10 and IL-35 Role in Developing Gestational Diabetes Mellitus.* Journal of Clinical and Diagnostic Research., 2020. **14**: p. OC01-OC03.

85. Khosrowbeygi, A., M.R. Rezvanfar, and H. Ahmadvand, *Tumor necrosis factor- alpha, adiponectin and their ratio in gestational diabetes mellitus.* Caspian J Intern Med, 2018. **9**(1): p. 71-79.

86. Mohammed, A. and I.S. Aliyu, *Maternal serum level of TNF-alpha in Nigerian women with gestational diabetes mellitus.* Pan Afr Med J, 2018. **31**: p. 250.

87. Mohammed, A., I.S. Aliyu, and M. Manu, *Correlation between circulating level of tumor necrosis factor-alpha and insulin resistance in Nigerian women with gestational diabetes mellitus.* Ann Afr Med, 2018. **17**(4): p. 168-171.

88. Zhou, X., et al., *Correlation of TNF-a, TNFR1 and adiponectin levels with HOMA-IR in patients with gestational diabetes mellitus.* 2017. **28**: p. 9549-9553.

89. Ertv , E.Y., et al. *Serum Lipid Profile and Inflammatory Status in Women with Gestational Diabetes Mellitus*. 2016.

90. Simon-Muela, I., et al., *Reduced circulating levels of TWEAK are associated with gestational diabetes mellitus.* Eur J Clin Invest, 2015. **45**(1): p. 27-35.

91. Li, J., et al., *Serum YKL-40 levels in gestational diabetes mellitus.* Gynecol Endocrinol, 2016. **32**(5): p. 412-5.

92. Giacobbe, A., et al., *Association between maternal serum high mobility group box 1 levels and pregnancy complicated by gestational diabetes mellitus.* Nutr Metab Cardiovasc Dis, 2016. **26**(5): p. 414-8.

93. Ye, X., et al., *Immune checkpoint molecule PD-1 acts as a novel biomarker for the pathological process of gestational diabetes mellitus.* Biomark Med, 2017. **11**(9): p. 741-749.

94. Pendeloski, K.P., et al., *Immunoregulatory molecules in patients with gestational diabetes mellitus.* Endocrine, 2015. **50**(1): p. 99-109.

95. Sahin Uysal, N., et al., *Is the serum delta neutrophil index level different in gestational diabetic women?* J Matern Fetal Neonatal Med, 2020. **33**(19): p. 3349-3354.

96. Ipekci, S.H., et al., *Serum levels of neopterin in gestational diabetes mellitus: the relationship with Apgar scores.* Arch Gynecol Obstet, 2015. **292**(1): p. 103-9.

97. Abbas, A., et al., *ASSOCIATION OF SERUM PREPTIN LEVELS WITH INSULIN RESISTANCE IN IRAQI WOMEN WITH GESTATIONAL DIABETES MELLITUS.* International research journal of pharmacy, 2019. **10**: p. 49-55.

98. Koroglu, N., et al., *Increased pancreatic-derived factor (PANDER) levels in gestational diabetes mellitus.* Gynecol Endocrinol, 2019. **35**(10): p. 866-868.

99. Adam, S., et al., *Association between gestational diabetes and biomarkers: a role in diagnosis.* Biomarkers, 2018. **23**(4): p. 386-391.

100. Zhu, C., et al., *Association of oxidative stress biomarkers with gestational diabetes mellitus in pregnant women: a case-control study.* PLoS One, 2015. **10**(4): p. e0126490.

101. Wang, Y.H., et al., *Changes of insulin resistance and beta-cell function in women with gestational diabetes mellitus and normal pregnant women during mid- and late pregnant period: a case-control study.* J Obstet Gynaecol Res, 2013. **39**(3): p. 647-52.

102. Saisho, Y., et al., *Beta cell dysfunction and its clinical significance in gestational diabetes.* Endocr J, 2010. **57**(11): p. 973-80.

103. Tan, P.C., et al., *Gamma-glutamyltransferase, alanine transaminase and aspartate transaminase levels and the diagnosis of gestational diabetes mellitus.* Clin Biochem, 2012. **45**(15): p. 1192-6.

104. Liu, H., et al., *Surrogate markers of the kidney and liver in the assessment of gestational diabetes mellitus and fetal outcome.* J Clin Diagn Res, 2015. **9**(1): p. OC14-7.

105. Nishimura, T., et al., *Lower serum total bilirubin concentration is associated with higher prevalence of gestational diabetes mellitus in Japanese pregnant women.* Endocr J, 2018. **65**(12): p. 1199-1208.

106. Zhao, W., et al., *Relationship between High Serum Cystatin C Levels and the Risk of Gestational Diabetes Mellitus.* PLoS One, 2016. **11**(2): p. e0147277.

107. Yousefzadeh, G., et al., *Plasma cystatin-C and risk of developing gestational diabetes mellitus.* Diabetes Metab Syndr, 2014. **8**(1): p. 33-5.

108. Winhofer, Y., et al., *CTX (crosslaps) rather than osteopontin is associated with disturbed glucose metabolism in gestational diabetes.* PLoS One, 2012. **7**(7): p. e40947.

109. Zhang, J., et al., *Characteristics of bone turnover markers in women with gestational diabetes mellitus.* Clin Biochem, 2020. **77**: p. 36-40.

110. Ural, U.M., et al., *Alteration of maternal serum irisin levels in gestational diabetes mellitus.* Ginekol Pol, 2016. **87**(5): p. 395-8.

111. Zhao, L., et al., *Circulating irisin is lower in gestational diabetes mellitus.* Endocr J, 2015. **62**(10): p. 921-6.

112. AL-Ghazali, *The Correlation of Irisin Levels and Some Trace Element as a Potential Mark Diagnosis of Gestational Diabetes Mellitus.* Acta Medica Iranica, 2019. **57**(1): p. 42-50.

113. Eschler, D.C., et al., *Circulating Levels of Bone and Inflammatory Markers in Gestational Diabetes Mellitus.* Biores Open Access, 2018. **7**(1): p. 123-130.

114. Talmor-Barkan, Y., et al., *Elevated galectin-3 in women with gestational diabetes mellitus, a new surrogate for cardiovascular disease in women.* PLoS One, 2020. **15**(6): p. e0234732.

115. Abdullah, B., et al., *Serum angiopoietin-related growth factor (AGF) levels are elevated in gestational diabetes mellitus and associated with insulin resistance.* Ginekol Pol, 2012. **83**(10): p. 749-53.

116. Dincgez Cakmak, B., et al., *Assessment of relationship between serum vascular adhesion protein-1 (VAP-1) and gestational diabetes mellitus.* Biomarkers, 2019. **24**(8): p. 750-756.

117. Bayoglu Tekin, Y., K. Baki Erin, and A. Yilmaz, *Evaluation of SCUBE-1 levels as a placental dysfunction marker at gestational diabetes mellitus.* Gynecol Endocrinol, 2020. **36**(5): p. 417-420.

118. Poniedzialek-Czajkowska, E., et al., *Intercellular Adhesion Molecule and Endogenous NOS Inhibitor: Asymmetric Dimethylarginine in Pregnant Women with Gestational Diabetes Mellitus.* J Diabetes Res, 2016. **2016**: p. 1342643.

119. Akturk, M., et al., *Asymmetric dimethylarginine concentrations are elevated in women with gestational diabetes.* Endocrine, 2010. **38**(1): p. 134-41.

120. Gomathi, K.G., et al. *Plasma Homocysteine and other Biochemical Parameters in Gestational Diabetes Mellitus*. 2011.

121. Artunc-Ulkumen, B., et al., *Maternal serum ADAMTS-9 levels in gestational diabetes: a pilot study.* J Matern Fetal Neonatal Med, 2017. **30**(12): p. 1442-1445.

122. Demir, E., et al., *Plasma Zonulin Levels as a Non-Invasive Biomarker of Intestinal Permeability in Women with Gestational Diabetes Mellitus.* Biomolecules, 2019. **9**(1).

123. Calan, M., et al., *The relationship between urotensin II and insulin resistance in women with gestational diabetes mellitus.* Hormones (Athens), 2019. **18**(1): p. 91-97.

124. Li, P., et al., *Plasma concentration of trimethylamine-N-oxide and risk of gestational diabetes mellitus.* Am J Clin Nutr, 2018. **108**(3): p. 603-610.

125. Mou, Y., et al. *Correlation of serum PEDF concentration with blood lipid in pregnant women with gestational diabetes mellitus*. 2016.

126. Bekdemir, H., et al., *Hemostatic changes in gestational diabetes mellitus.* International Journal of Diabetes in Developing Countries, 2015. **35**(3): p. 502-506.

127. Gorkem, U., C. Togrul, and E. Arslan, *Relationship between elevated serum level of placental growth factor and status of gestational diabetes mellitus.* J Matern Fetal Neonatal Med, 2020. **33**(24): p. 4159-4163.

128. Theriault, S., et al., *Early prediction of gestational diabetes: a practical model combining clinical and biochemical markers.* Clin Chem Lab Med, 2016. **54**(3): p. 509-18.

129. B.A., S., *Biochemical Markers Predicting the Risk of Gestational Diabetes Mellitus.* Materiale Plastice, 2017. **54**: p. 133-136.

130. Melekoglu, R., et al., *Evaluation of second trimester amniotic fluid ADAMTS4, ADAMTS5, interleukin-6 and tumor necrosis factor-alpha levels in patients with gestational diabetes mellitus.* J Obstet Gynaecol Res, 2019. **45**(4): p. 824-829.

131. Nar, G., et al., *The relationship between epicardial fat thickness and gestational diabetes mellitus.* Diabetol Metab Syndr, 2014. **6**(1): p. 120.

132. D'Ambrosi, F., et al., *Maternal Subcutaneous and Visceral Adipose Ultrasound Thickness in Women with Gestational Diabetes Mellitus at 24-28 Weeks' Gestation.* Fetal Diagn Ther, 2018. **43**(2): p. 143-147.

133. Kansu-Celik, H., et al., *Relationship maternal subcutaneous adipose tissue thickness and development of gestational diabetes mellitus.* Interv Med Appl Sci, 2018. **10**(1): p. 13-18.

134. Tosun, A., et al., *Utility of superior mesenteric artery Doppler and maternal pancreatic size for predicting gestational diabetes mellitus.* Ir J Med Sci, 2015. **184**(2): p. 499-503.

135. Yousefzadeh, G., et al., *Increased carotid artery intima-media thickness in pregnant women with gestational diabetes mellitus.* J Tehran Heart Cent, 2012. **7**(4): p. 156-9.

136. Bugatto, F., et al., *The Influence of Lipid and Proinflammatory Status on Maternal Uterine Blood Flow in Women With Late Onset Gestational Diabetes.* Reprod Sci, 2018. **25**(6): p. 837-843.

137. Meera, S.J., et al., *Dynamic left ventricular changes in patients with gestational diabetes: A speckle tracking echocardiography study.* J Clin Ultrasound, 2017. **45**(1): p. 20-27.

138. Soydinc, H.E., et al., *Circadian variation of blood pressure is impaired in normotensive pregnant women with gestational diabetes mellitus.* Clin Exp Hypertens, 2013. **35**(2): p. 128-33.

139. Sovio, U., H.R. Murphy, and G.C. Smith, *Accelerated Fetal Growth Prior to Diagnosis of Gestational Diabetes Mellitus: A Prospective Cohort Study of Nulliparous Women.* Diabetes Care, 2016. **39**(6): p. 982-7.

140. Akiba, Y., et al., *Differences in fetal fractional limb volume changes in normal and gestational diabetic pregnancies: an exploratory observational study.* BJOG, 2021. **128**(2): p. 329-335.

141. Jin, D., et al., *Gestational Diabetes Mellitus: Predictive Value of Fetal Growth Measurements by Ultrasonography at 22-24 Weeks: A Retrospective Cohort Study of Medical Records.* Nutrients, 2020. **12**(12).

142. Ilhan, G., et al., *Preliminary evaluation of foetal liver volume by three-dimensional ultrasound in women with gestational diabetes mellitus.* J Obstet Gynaecol, 2018. **38**(7): p. 922-926.

143. Showman, H.A.K., H.A.G. Al-Rawi, and M.A.G. Zghair, *The value of mid-trimester fetal liver length measurement in prediction of gestational diabetes in Iraqi women*. 2020. Vol. 25. 2020.

144. Venkataraman, H., et al., *Increased fetal adiposity prior to diagnosis of gestational diabetes in South Asians: more evidence for the 'thin-fat' baby.* Diabetologia, 2017. **60**(3): p. 399-405.

145. Aksoy, H., et al., *Fetal anterior abdominal wall thickness may be an early ultrasonographic sign of gestational diabetes mellitus.* J Matern Fetal Neonatal Med, 2016. **29**(12): p. 2028-32.

146. Tantanasis, T., et al., *Sonographic assessment of fetal subcutaneous fat tissue thickness as an indicator of gestational diabetes.* Eur J Obstet Gynecol Reprod Biol, 2010. **152**(2): p. 157-62.

147. Yavuz, A., et al., *Second Trimester Fetal and Maternal Epicardial Fat Thickness in Gestational Diabetic Pregnancies.* Horm Metab Res, 2016. **48**(9): p. 595-600.

148. Aydin, S. and E. Fatihoglu, *Fetal Epicardial Fat Thickness: Can It Serve as a Sonographic Screening Marker for Gestational Diabetes Mellitus?* J Med Ultrasound, 2020. **28**(4): p. 239-244.

149. Aguilera, J., et al., *Paired maternal and fetal cardiac functional measurements in women with gestational diabetes mellitus at 35-36 weeks' gestation.* Am J Obstet Gynecol, 2020. **223**(4): p. 574 e1-574 e15.

150. Abdel Gader, A.G., et al., *Haemostatic and cytokine changes in gestational diabetes mellitus.* Gynecol Endocrinol, 2011. **27**(5): p. 356-60.

151. Jatavan, P., et al., *The correlation of fetal cardiac function with gestational diabetes mellitus (GDM) and oxidative stress levels.* J Perinat Med, 2020. **48**(5): p. 471-476.

152. Dantas, A.M.A., et al., *Doppler ultrasonographic assessment of fetal middle cerebral artery peak systolic velocity in gestational diabetes mellitus.* Int J Gynaecol Obstet, 2019. **144**(2): p. 174-179.

153. To, W.W. and C.K. Mok, *Fetal umbilical arterial and venous Doppler measurements in gestational diabetic and nondiabetic pregnancies near term.* J Matern Fetal Neonatal Med, 2009. **22**(12): p. 1176-82.

154. Najafi, L., et al., *Antenatal umbilical coiling index in gestational diabetes mellitus and non-gestational diabetes pregnancy.* Taiwan J Obstet Gynecol, 2018. **57**(4): p. 487-492.

155. Bildaci, T.B., et al., *Placental elasticity on patients with gestational diabetes: Single institution experience.* J Chin Med Assoc, 2017. **80**(11): p. 717-720.

156. Suranyi, A., et al., *Placental three-dimensional power Doppler indices in mid-pregnancy and late pregnancy complicated by gestational diabetes mellitus.* Prenat Diagn, 2013. **33**(10): p. 952-8.

157. Perovic, M., et al., *Sensitivity and specificity of ultrasonography as a screening tool for gestational diabetes mellitus.* J Matern Fetal Neonatal Med, 2012. **25**(8): p. 1348-53.

158. Taricco, E., et al., *Effects of gestational diabetes on fetal oxygen and glucose levels in vivo.* BJOG, 2009. **116**(13): p. 1729-35.

159. Teng, F., et al., *Expression of intercellular adhesion molecule-1 in umbilical vascular of pregnant women with gestational diabetes mellitus and the clinical significance.* Exp Ther Med, 2018. **15**(1): p. 914-918.

160. Zhang, Y., et al., *Serum levels of nesfatin-1 are increased in gestational diabetes mellitus.* Gynecol Endocrinol, 2017. **33**(8): p. 621-624.

161. Aslan, M., et al., *Cord blood nesfatin-1 and apelin-36 levels in gestational diabetes mellitus.* Endocrine, 2012. **41**(3): p. 424-9.

162. Shang, M., X. Dong, and L. Hou, *Correlation of adipokines and markers of oxidative stress in women with gestational diabetes mellitus and their newborns.* J Obstet Gynaecol Res, 2018. **44**(4): p. 637-646.

163. Karakulak, M., et al., *Comparison of umbilical cord ghrelin concentrations in full-term pregnant women with or without gestational diabetes.* Endocr Res, 2017. **42**(2): p. 79-85.

164. Qi, X., et al., *Decreased cord blood estradiol levels in related to mothers with gestational diabetes.* Medicine (Baltimore), 2017. **96**(21): p. e6962.

165. Celik, E., et al., *Maternal and fetal adropin levels in gestational diabetes mellitus.* J Perinat Med, 2013. **41**(4): p. 375-80.

166. Dube, M.C., et al., *Cord blood C-peptide levels relate to the metabolic profile of women with and without gestational diabetes.* Acta Obstet Gynecol Scand, 2012. **91**(12): p. 1469-73.

167. McManus, R., et al., *Maternal, umbilical arterial and umbilical venous 25-hydroxyvitamin D and adipocytokine concentrations in pregnancies with and without gestational diabetes.* Clin Endocrinol (Oxf), 2014. **80**(5): p. 635-41.

168. Barke, T.L., et al., *Gestational diabetes mellitus is associated with increased CD163 expression and iron storage in the placenta.* Am J Reprod Immunol, 2018. **80**(4): p. e13020.

169. Usta, A., et al., *Expression of cyclophilin A in the placental tissue of pregnant women with gestational diabetes mellitus.* Analytical and Quantitative Cytopathology and Histopathology, 2017. **39**: p. 238-246.

170. Navarro, A., et al., *Increase in placental apolipoprotein D as an adaptation to human gestational diabetes.* Placenta, 2010. **31**(1): p. 25-31.

171. Dairi, A.S., et al., *The Effect of Gestational Diabetes Mellitus on the Chorionic Villi of Human Placenta Among Saudi Arabian Mothers: A Quantitative and Comparative Study.* Cureus, 2020. **12**(10): p. e11130.

172. Kadivar, M., et al., *Histomorphological changes of the placenta and umbilical cord in pregnancies complicated by gestational diabetes mellitus.* Placenta, 2020. **97**: p. 71-78.

173. Pooransari, P., et al., *Is gross morphology of placenta, umbilical cord, and neonatal outcome in well-controlled gestational diabetes mellitus pregnancy different? A case-control study.* Int J Reprod Biomed, 2020. **18**(6): p. 407-414.

174. Kucuk, M. and F. Doymaz, *Placental weight and placental weight-to-birth weight ratio are increased in diet- and exercise-treated gestational diabetes mellitus subjects but not in subjects with one abnormal value on 100-g oral glucose tolerance test.* J Diabetes Complications, 2009. **23**(1): p. 25-31.
