# Supplementary material for: The diagnostic indicators of gestational diabetes mellitus from second trimester to birth: a systematic review
Source: Clin Diabetes Endocrinol. 2021 Oct 11;7:19. doi: 10.1186/s40842-021-00126-7 (PMC8504031; doi:10.1186/s40842-021-00126-7)
Supplement: Supplementary file 3 — Additional file 3. Table used to extract data from the articles assessed [file 40842_2021_126_MOESM3_ESM.docx]

**Additional file 3. Data extraction form**

| Author Year and Title | Country of study | Aim | Pts I/E criteria * | GDM Diagnosis | Cases / controls n. | GA | Biomarker | Research Design | Methods | Main Findings | Conclusions | CASP score |
| --- | --- | --- | --- | --- | --- | --- | --- | --- | --- | --- | --- | --- |
|  |  |  |  |  |  |  |  |  |  |  |  |  |
|  |  |  |  |  |  |  |  |  |  |  |  |  |
|  |  |  |  |  |  |  |  |  |  |  |  |  |
|  |  |  |  |  |  |  |  |  |  |  |  |  |
|  |  |  |  |  |  |  |  |  |  |  |  |  |
|  |  |  |  |  |  |  |  |  |  |  |  |  |

GA= Gestational Age,

*Patients Inclusion/Exclusion criteria
