# Supplementary material for: The diagnostic indicators of gestational diabetes mellitus from second trimester to birth: a systematic review
Source: Clin Diabetes Endocrinol. 2021 Oct 11;7:19. doi: 10.1186/s40842-021-00126-7 (PMC8504031; doi:10.1186/s40842-021-00126-7)
Supplement: Supplementary file 4 — Additional file 4. Table of all the biomarkers evaluated divided into category and described with their name, abbreviation, function. [file 40842_2021_126_MOESM4_ESM.docx]

**Additional file 4. Biomarkers evaluated**

| **NAME** | **SIGLE** | **CATEGORY** | **FUNCTION/SIGNIFICANCE** |
| --- | --- | --- | --- |
| *Fructosamine* | N.A. | Advanced glycation end products | Reflects albumin glycation in the last 2 weeks |
| *Glycated albumin* | GA | Advanced glycation end products | Albumin modulator of plasma oncotic pressure and transporter |
| *Glycated haemoglobin* | HbA1c | Advanced glycation end products | HbA1 modulator of erythrocyte metabolism |
| N*(epsilon)-(*carboxymethyl*)*lysine*,* | CML | Advanced glycation end products | Produced by oxidation of fructosyl-lysine |
| *Plasma glycated CD59* | pGCD59 | Advanced glycation end products | CD59 Compliment Inhibitor |
| *2-Aminobutyric Acid* | N.A. | Amino Acid | Protective Effects Against Oxidative Stress |
| *Arginine, Glycine and Methionine* | ARG, GLY, MET | Amino acid | Combine to form proteins |
| *Homocysteine* | HCYS | Amino acid | Associated with atherosclerosis |
| *Activin A* | Act-A | Cytokine | Member of the transforming growth factor beta (TGF-β) family - [Cell proliferation](https://www.sciencedirect.com/topics/medicine-and-dentistry/cell-proliferation), differentiation, wound healing, [apoptosis](https://www.sciencedirect.com/topics/medicine-and-dentistry/programmed-cell-death), and metabolism |
| *Angiopoietin-Related Growth Factor aka or angiopoietin-like protein-6* | *AGF/*ANGPTL6 | Cytokine | Stimulates metabolism and angiogenesis |
| *Apelin- 36* | N.A. | Cytokine | Related to the nutritional status and parallel insulin plasma levels |
| *B-cell activating factor* | BAFF | Cytokine | Positive regulator of B-cell function and expansion |
| *Chemerin* | N.A. | Cytokine | Adipokine: Involved in inflammation, adipogenesis, angiogenesis and energy metabolism |
| *Coiled-coil domain-containing 80* | CCDC80 | Cytokine | Increased in obesity |
| *Follistatin-like-3* | FSTL3 | Cytokine | Activin antagonist |
| *Interleukin - 6* | IL6 | Cytokine | Proinflammatory action |
| *Interluekin-10* | Il-10 | Cytokine | Anti-inflammatory |
| *Interleukin-18 (aka interferon-gamma inducing factor)* | IL-18 | Cytokine | Proinflammatory action |
| *Interleukin - 35* | IL-35 | Cytokine | Proinflammatory action |
| *Irisin* | N.A. | Cytokine | Influences metabolism |
| *Pancreatic-Derived Factor* | PANDER | Cytokine | Regulates the process of insulin release and glucose homeostasis |
| *Platelet-activating factor* | PAF | Cytokine | Stimulates platelet aggregation, vasodilation, inflammation |
| *Retinol-binding protein 4* | RBP4 | Cytokine | Adipokine: promotes insulin resistance |
| *Tumour necrosis factor A* | TNF-A | Cytokine | Proinflammatory action |
| *Tumour necrosis factor-like weak inducer of apoptosis* | TWEAK | Cytokine | Proangiogenic and proinflammatory properties |
| *Growth differentiation factor 15* | GDF-15 | Growth Factor | Regulates response to injury |
| *Hepatocyte Growth Factor* | HGF | Growth Factor | Produced by liver and placental tissues promotes cell survival and tissue regeneration in liver, pancreas, kidneys heart and placenta. |
| *Placental Growth Factor* | PlGF | Growth Factor | Placental development: angiogenesis and trophoblastic invasion of the maternal spiral arteries |
| *Adipocyte fatty acid binding protein* | AFABP | Hormone | Promotes insulin resistance |
| *Aprosin* | N.A. | Hormone | Produced in white adipose tissues, stimulates the liver to release glucose into the blood stream |
| *Atrial natriuretic peptide* | ANP | Hormone | Decrease in [systemic](https://en.wikipedia.org/wiki/Systemic_circulation) [vascular resistance](https://en.wikipedia.org/wiki/Vascular_resistance) and [central venous pressure](https://en.wikipedia.org/wiki/Central_venous_pressure) as well as an increase in [natriuresis](https://en.wikipedia.org/wiki/Natriuresis) |
| *Brain-type natriuretic peptide I* | BNP - l | Hormone | Same as ANP but 10-fold lower affinity for the receptor and twice [biological half-life](https://en.wikipedia.org/wiki/Biological_half-life) |
| *Betatrophin* | N.A. | Hormone | Controls B cells proliferation |
| *(Unconjugated) Estriol* | UE | Hormone | Free quote of Estriol, poor stimulator of uterine growth and plasminogen activator activity |
| *Ghrelin* | N.A. | Hormone | Regulation of eating behaviour and body weight |
| *Insulin* | N.A. | Hormone | Facilitates cellular glucose uptake, regulates metabolism/promotes cell division and growth |
| *Leptin* | N.A. | Hormone | Produced by adipose cells and enterocytes inhibits hunger |
| *Prolactin* | PRL | Hormone | Promotes and sustains lactation and glucose homeostasis |
| *Preptin* | N.A. | Hormone | Co-secreted with insulin and amylin from the pancreatic beta-cells, increases glucose-mediated insulin secretion |
| *Resistin (AKA adipose tissue-specific secretory factor or C/EBP-epsilon-regulated myeloid-specific secreted cysteine-rich protein)* | ADSF/ XCP1 | Hormone | [Adipose derived](https://en.wikipedia.org/wiki/Adipose-derived_hormone) involved in obesity and T2DM development |
| **NAME** | **SIGLE** | **CATEGORY** | **FUNCTION/SIGNIFICANCE** |
| *Thyroid Hormones: Thyroid Stimulating Hormone Triiodothyronine Thyroxine* | TSH FT3 FT4 | Hormone | . Stimulates the thyroid to produce T4/T3 Active form– activate the metabolism Transformed into T3 |
| *Lysophospholipids (Lysophosphatidylethanolamine-LPE; Lysophosphatidylcholine-LPC; Lysophosphatidylinositol – LPI; Lysophosphoserune- LPS; Lysophosphatidic acid –LPA)* | LPLs | Lipid molecules | Extra/intra-cellular mediators |
| *Triglycerides* | N.A. | Lipid molecules | Fat storage |
| *Asymmetric Dimethylarginine* | ADMA | Others | *Enzyme* Inhibitor: Inhibit NO synthesis impairing endothelial function and promoting atherosclerosis |
| *Bile Acids (dihydroxy conjugated, trihydroxy unconjugated and sulfated bile acids)* | N.A. | Others | Lipid derived product : Facilitate [digestion of dietary fats and oils](https://en.wikipedia.org/wiki/Digestion#Fat_digestion) |
| *3-carboxy-4-methyl-5-propyl-2-furanpropanoic acid* | CMPF | Others | Organic compound : Uremic toxin |
| *Disulfide* | N.A. | Others | Protein component: Disulfide bonds play a key role in stabilizing protein structures, with disruption strongly associated with loss of protein function and activity due to oxidation |
| *Endogenous NOS Inhibitor* | N.A. | Others | *Enzyme* Inhibitor : Contributes to endothelial dysfunction |
| *Iron* | N.A. | Others | Element: Haemoglobin and Myoglobin production |
| *Itaconic acid* | IA | Others | Organic compound : Possess antibacterial activities |
| *Long-chain polyunsaturated fatty acids (including omega-3 (docosahexaenoic acid or DHA)* | (LCPUFAs) | Others | Organic compound : Required for normal growth, to support immunity, and can improve cardiovascular and brain health. |
| *Neopterin* | N.A. | Others | Nucleotide derived: Marker for the activation of the immune system and neuroinflammation |
| *T Lymphocytes* | *CD4, CD69, And CD8 T* | Others | Cells: Components of the adaptive immune system |
| *Total Bilirubin* | TB | Others | Lipid derived Pigment : Shown to possess important functions as an antioxidant, it allows the excretion of heme, from haemoglobin, myoglobin, and various P450 enzymes. |
| *8 Isoprostane (of which 8-iso-prostaglandin (8-iso-PGF2𝛼),)* | 8Isop | Oxidation/ Peroxidation product | Marker of oxidative stress |
| *Malondialdehyde* | MDA | Oxidation/ Peroxidation Product | Marker of oxidative stress |
| *Methylglyoxal* | MGO | Oxidation/ Peroxidation Product | Oxidizing substance leades to oxidative stress, cellular aging, DNA mutations, and apoptosis |
| *Nitrotyrosine* | NT | Oxidation/ Peroxidation product | Marker of cell damage, inflammation as well as NO (nitric oxide) production. |
| *Oxidised low-density lipoprotein* | Ox-LDL | Oxidation/ Peroxidation Product | Atherosclerosis |
| *Protein Carbonyl* | PCO | Oxidation/ Peroxidation product | Markers of oxidative stress |
| *Protein Hydroperoxides* | P-OOH | Oxidation/ Peroxidation Product | Direct reactivity with a variety of biomolecules and the ability to decompose to free radicals |
| *Trimethylamine-N-Oxide* | TMAO | Oxidation/ Peroxidation product | Independent risk factor for the development of atherosclerosis and cardiovascular diseases |
| *Adropin* | N.A. | Peptide | Maintenance of energy homeostasis and insulin secretion |
| *β-C-terminal telopeptide of type 1 collagen* | β-CTX | Peptide | Marker of bone resorption |
| *Copeptin* | CT-proAVP | Peptide | Vasopressin surrogate marker |
| *C-peptide* | N.A. | Peptide | reflects the insulin-secretory activity of pancreatic *β*-cells |
| *C-Terminal Cross-Linking Telopeptide Of Type-I Collagen* | CTX | Peptide | Marker of bone resorption |
| *Galanin* | N.A. | Peptide | Neuronal inhibitor |
| *Nesfatin-1* | N.A. | Peptide | Regulation of hunger and fat storage |
| *N-terminal midfragment of osteocalcin* | N-MID | Peptide | Marker of bone formation |
| *Procollagen type 1 N-terminal propeptide* | P1NP | Peptide | Marker of bone formation |
| *Spexin* | N.A. | Peptide | Involvement in energy homeostasis and food intake |
| *Urotensin II* | UII | Peptide | Modulation of vessels dynamics and insulin resistance |
| *Adiponectin* | N.A. | Protein | Adipocyte-specific involved in insulin resistance and atherosclerosis |
| **NAME** | **SIGLE** | **CATEGORY** | **FUNCTION/SIGNIFICANCE** |
| *A Disintegrin And Metalloproteinase With Thrombospondin Motifs 9* | ADAMTS-9 | Protein | Extracellular matrix re-modelling, angiogenesis, fibrosis, and coagulation |
| *Apolipoprotein A1* | ApoA1 | Protein | Primary protein component of high-density lipoprotein (HDL) |
| *Apolipoprotein D* | apo D | Protein | Antioxidant |
| *Chitinase-3 like-protein-1* | YKL-40 | Protein | Marker of inflammation - binds to chitin, heparin, and hyaluronic acid |
| *Cluster of Differentiation 163* | Cd163 | Protein | Haemoglobin scavenger receptor is a macrophage specific protein characteristic of tissues responding to inflammation |
| *C reactive Protein* | CRP | Protein | Marker of inflammation |
| *Cyclophilin A* | CyPA | Protein | Mediates intracellular protein folding, intracellular and extracellular trafficking, and protein-protein interaction; immunosuppressive effect. |
| *Cystatin C* | Cys-C | Protein | Marker of glomerular filtration |
| *Ficolin 3* | FCN 3 | Protein | Activates the complement pathway |
| *Ferritin* | N.A. | Protein | Iron storage |
| *Fibrinogen aka Factor I* | N.A. | Protein | Involved coagulation, revascularization and [wound healing](https://en.wikipedia.org/wiki/Wound_healing) |
| *Follistatin* | FS | Protein | Binding and bio neutralization of members of the TGF-β superfamily |
| *Galectin-3* | Gal-3 | Protein | Mediator of cell damage: pro-fibrotic and pro-inflammatory properties |
| *Glutathione Peroxidase-3* | GPX-3 | Protein | Antioxidant |
| *High Mobility Group Box 1* | HMGB1 | Protein | Nuclear protein that organizes the DNA and regulates transcription |
| *Human Cartilage Glycoprotein 39* | YKL-40 | Protein | Plays important roles in inflammation, extracellular remodelling, fibrosis, and angiogenesis |
| *Neuregulin 4* | NRG4 | Protein | Signalling |
| *Osteopontin* | OPN | Protein | Involved in physiological and pathological bone mineralization and inflammatory disorders |
| *Osteoprotegerin aka Osteoclastogenesis inhibitory factor or Tumour necrosis factor receptor superfamily member 11B* | OPG/OCIF/TNFRSF11B | Protein | Regulates bone density and inhibits apoptosis of specific cells |
| *Paraoxonase-1* | PON1 | Protein | Anti-inflammatory & Antioxidant |
| *Pigment Epithelium Derived Factor* | PEDF | Protein | Related to insulin sensitivity and involved in the occurrence and complications of diabetes mellitus (nephropathy and retinopathy) |
| *Plasminogen* | N.A. | Protein | Plasmin precursor |
| *Plasminogen Activator Inhibitor Type 1* | PAI-1 | *Protein* | Involved in tumorigenesis, angiogenesis, wound healing, ovulation, and regulation of anti-fibrinolytic activity of the plasma. |
| *Pre-Albumin (transthyretin)* |  | Protein | Carries thyroxine (the main thyroid hormone) and vitamin A |
| *Protein convertase subtilisin/kexin* | PCSK | Protein | Induce degradation of LDL receptors in the lysosome of hepatocytes reducing ldl metabolism and generating inflammation |
| *Programmed Cell Death Protein 1* | PD-1/ CD279 | Protein | Down-regulates the immune system and promotes self-tolerance by suppressing T cell |
| *Receptor Activator Of Nuclear Factor-Kappa B Ligand aka Osteoprotegerin ligand* | RANKL/ OPGL | Protein | Stimulates osteoclast differentiation and activity, as well as prevention of osteoclast apoptosis |
| *Secreted frizzled-related protein 4* | SFRP4 | Protein | Regulates bone morphogenesis/ adult uterine morphology and function/ apoptosis during ovulation |
| *Sex hormone binding globulin* | SHBG | Protein | Hormones transport |
| *Signal peptide-CUB-EGF domain-containing protein* | SCUBE-1 | Protein | Expression of hypoxia, endothelial dysfunction, and vascular injury |
| *Soluble Adhesion Molecules* | sICAM-1, sVCAM-1 | Protein | Influence the binding of monocytic cells to vascular endothelium in inflammatory processes |
| *TNF-a soluble receptors* | sTNFaRs: sTNF-R1 and sTNF-R2 | Protein | Their concentration is proportional to previous TNFa action and remain elevated in plasma for longer periods |
| *Transferrin* | TfR | Protein | Iron metabolism |
| *Transferrin receptor* | sTfR | Protein | Cellular iron acquisition |
| *Transthyretin* | TTR | Protein | Transport protein in serum/cerebrospinal fluid for thyroxine (T_4_) and retinol-binding protein bound to retinol |
| *Tumor necrosis factor-related apoptosis-inducing ligand* | TRAIL | Protein | Expressed on the surface of natural killer and T cells, macrophages, and dendritic cells. |
| *Vascular adhesion protein 1* | VAP-1 | *Protein* | Surface adhesion molecule, mediates leukocyte extravasation and contributes to oxidative stress |
| *Visceral adipose tissue derived serine [protease](https://www.sciencedirect.com/topics/pharmacology-toxicology-and-pharmaceutical-science/proteinase) inhibitor (aka Serpin A12)* | VASPIN | Protein | Improves glucose tolerance and reduces food intake |
| *Von Willerbrand Factor* | VWF | Protein | Coagulation |
| *Zonulin* | N.A. | Protein | Biomarker of impaired gut barrier function for several autoimmune, neurodegenerative, and tumoral diseases |
| *Adiponectin/TNF-α ratio* | N.A. | Score/ratio/index | Expression of anti vs pro inflammatory molecules |
| *Body Mass Index* | BMI | Score/ratio/index | =weight/height^2^ |
| *Delta Neutrophil Index* | DNI | Score/ratio/index | Corresponds to the fraction of circulating immature granulocytes, linked to infection |
| **NAME** | **SIGLE** | **CATEGORY** | **FUNCTION/SIGNIFICANCE** |
| *Disposition Index* | DI | Score/ratio/index | Product of insulin sensitivity times the amount of insulin secreted in response to blood glucose levels. |
| *Fasting Glucose (Plasma/Blood)* | FG – FPG/FBG | Score/ratio/index | Level of blood glucose at fasting |
| *HOMA pancreatic β-cell function* | HOMA-B | Score/ratio/index | Estimates steady state beta cell function (%B) as percentages of a normal reference population |
| *Homeostatic model assessment for Insulin resistance - index* | HOMA-IR | Score/ratio/index | Resistance index derived by fasting glucose and insulin |
| *Insulin sensitivity index* | ISI | Score/ratio/index | Function of the measured glucose and insulin levels |
| *Insulinogenic index* | IGI | Score/ratio/index | Index of insulin secretion derived from OGTT = δ insulin (0-30 min)/δ glucose (0-30 min) |
| *Neutrophil-to-lymphocyte ratio and* | NLR | Score/ratio/index | Marker of inflammation |
| *Platelet-to-lymphocyte ratio* | PLR | Score/ratio/index | Marker of inflammation |
| *Quantitative Insulin Sensitivity Check* | QUICKI | Score/ratio/index | = 1/[log(I_0_) + log(G_0_)] where I_0_ is the fasting insulin, and G_0_ is the fasting glucose |
| *Serum Transferrin/ferretin ratio* | sTfR-F ratio | Score/ratio/index | Covers the full spectrum of iron homeostasis, from normal, healthy iron stores to mild or substantial functional iron deficiency |
| *Total Antioxidant Capacity* | TAC | Score/ratio/index | Measure of the number of free radicals scavenged by a test solution |
| *Transferrin saturation* | TS | Score/ratio/index | Serum iron/ total iron-binding capacity of available transferrin (TIBC) |
| *Triglyceride to high-density lipoprotein cholesterol ratio* | TG/HDL-C | Score/ratio/index | High levels associated with obesity, metabolic syndrome, and insulin resistance |
| *Triglyceride-glucose index* | TyG | Score/ratio/index | Insulin resistance Index |
| *Butyrylcholinesterase Activity* | BChE activity | Score/ratio/index – Miscellaneous markers | Used as a liver function test |
| *Disuplhide/Native Thiol,* | N.A. | Score/ratio/index – Miscellaneous markers | Expression of oxidation |
| *Disulfide/Total Thiol* | N.A. | Score/ratio/index – Miscellaneous markers | Expression of oxidation |
| *High-sensitivity C-reactive protein* | Hs-CRP | Score/ratio/index – Miscellaneous markers | Finds lower levels of CRP - Cardiovascular disease |
| *Isovolumic Relaxation Time* | IVRT | Score/ratio/index – Miscellaneous markers | Time interval between the end of aortic ejection and the beginning of ventricular filling in ms. |
| *Mean Platelet Volume* | MPV | Score/ratio/index – Miscellaneous markers | Average platelet size |
| *Native thiol/total thiol ratio* | N.A. | Score/ratio/index – Miscellaneous markers | Expression of oxidation |
| *Oxidative Stress Index* | OSI | Score/ratio/index – Miscellaneous markers | Ratio of TOS to TAS (Total antioxidant status) |
| *Placental barrier thickness/surface area of the blood vessels ratio* | N.A. | Score/ratio/index – Miscellaneous markers | Adaptation to diabetes-related hypoxia |
| *Placental Hoffbauer cells n /surface area of the villous ratio* | N.A. | Score/ratio/index – Miscellaneous markers | Adaptation to diabetes-related hypoxia |
| *Placental Surface area of the blood vessel,* | N.A. | Score/ratio/index – Miscellaneous markers | Adaptation to diabetes-related hypoxia |
| *Placental weight/birth weight ratio (PW/BW)* | N.A. | Score/ratio/index – Miscellaneous markers | Reflex of the growth-promoting environment of GDM |
| *Tei index* | N.A. | Score/ratio/index – Miscellaneous markers | Marker of cardiac function: sum of the isovolumic contraction and relaxation times divided by the ejection time |
| *Total Oxidative Stress* | TOS | Score/ratio/index – Miscellaneous markers | Marker of the overall oxidation state of the body |
| *Umbilical coiling index* | UCI | Score/ratio/index – Miscellaneous markers | Total number of coils divided by the total length of the cord |
| **NAME** | **SIGLE** | **CATEGORY** | **FUNCTION/SIGNIFICANCE** |
| *Estimated fetal weight* | EFW | Ultrasound measure | Derived with Hadlock formula: HC, AC, and FL |
| *Femur length* | FL | Ultrasound measure | Basic biometric parameter used to assess fetal size |
| *Epicardial Fat Thickness* | EFT | Ultrasound measure | Echo-free space between the outer wall of the myocardium and the visceral layer of the pericardium |
| *Fetal Liver length* | FLL | Ultrasound measure | Measure of the fetal liver |
| *Fractional arm volume* | AVol | Ultrasound measure | Cylindrical limb volume based on 50% of the fetal  humeral diaphysis length |
| *Fractional thigh volume* | TVol | Ultrasound measure | Cylindrical limb volume based on 50% of the fetal  femoral diaphysis length |
| *Head circumference* | HC | Ultrasound measure | Basic biometric parameter used to assess fetal size |
| *Cobalamin* | Vit B12 | Vitamin | Involved in cells metabolism |
| *Vitamin D* | 25(OH)D | Vitamin | Calcium metabolism and anti-inflammatory action |

N.A.=Not available
